# Effects of Ginger and Garlic Powders on the Physicochemical and Microbiological Characteristics of Fruit Juices during Storage

**DOI:** 10.3390/foods12061311

**Published:** 2023-03-19

**Authors:** Ancuța Elena Prisacaru, Cristina Ghinea, Eufrozina Albu, Florin Ursachi

**Affiliations:** 1 Faculty of Food Engineering, Stefan cel Mare University of Suceava, 720229 Suceava, Romania; 2 Suceava-Botoșani Regional Innovative Bioeconomy Cluster Association, 720229 Suceava, Romania

**Keywords:** apple, juice, natural preservatives, pumpkin, pomegranate, storage

## Abstract

Natural preservatives such as garlic and ginger can be added to the formulation of fresh fruit juices to encourage the consumption of health-promoting foods. In this study, the influence of garlic and ginger and the storage conditions on physicochemical and microbiological characteristics of fruit juices were investigated. The fruit juice assortments were produced from apple, apple and pumpkin, and apple and pomegranate and were treated with 0.5 g garlic powder, 0.5 g ginger powder, and 0.25 g mix of garlic and ginger powders. A total of 12 unpasteurized samples were produced, of which 3 were control samples. Samples stored at 20 and 4 °C were analyzed at 0, 3, 6, and 9 days for water activity (aw), pH, titratable acidity (TA), total soluble solids (TSS), electrical conductivity (*EC*), vitamin C, color parameters, total number of germs, yeasts, and molds, Listeria, Enterobacteriaceae, and *Escherichia coli*. Results showed that aw, pH, TSS, and vitamin C content decreased during storage of fruit juice samples, while TA increased. The lowest increase in total number of aerobic mesophilic germs was determined for the apple and pumpkin juice with garlic and ginger and apple juice with garlic.

## 1. Introduction

Fruit and vegetable consumption plays an important role in people’s nutrition and health [1], being associated with a reduced risk of chronic diseases and body weight management [2,3].

Apple (*Malus domestica*) is one of the fruits that is produced in large quantities all over the world [4]. In the 2021/2022 harvest year, approximately 46 million metric tons of apples were produced in China alone and 12.28 million metric tons of apples in the European Union according to Statista [5]. The largest producer of apples in the European Union is Poland [1], while Romania is in fourth place and produced approximately 11.75% of the total apple production in 2020 [6]. Depending on the variety, apples can have a moisture content between 83.97 and 86.27%, a water activity between 0.906 and 0.910, and an average pH between 4.02 and 4.19. The total solids content of fresh apple fruit can vary between 15.00 and 16.46 °Brix, while electrical conductivity values can vary between 548 and 1854 µS/cm [4]. Additionally, apple fruits contain sugar (7.41–14.2%), malic acid (0.041–2.97%), total extractable phenolic content (0.11–0.374%), hydroxycinnamic acid (2.52–93.6 mg/kg), and flavanols (15–398 mg/kg) [1]. The consumption of apple fruit has significant beneficial effects on health: the ability to reduce cholesterol and slow down the absorption of glucose, protect against cardiovascular diseases, and have anti-inflammatory and antioxidant effects [7]. Apples are consumed fresh and processed in the form of juice, cider, vinegar, sweets, jams, and others [1].

Pumpkin (*Cucurbita* spp.) is available in different shapes, sizes, and colors [8] and approximately 23 million tons were produced worldwide in 2019, with China, the United States, India, and Russia as the main producers [8,9]. In the European Union, 760,000 tons were harvested in 2020, and 85% of this amount was produced by Poland, Spain, France, Portugal, and Germany. In the same year, 21,260 tons of pumpkins were produced in Romania [10]. Pumpkin contains dietary fiber, carotenoids, carbohydrates, aspartic acid, glutamic acid, arginine, calcium, iron, zinc, and copper [8]. The pumpkin pulp is characterized by a pH between 4.27 and 7.79, a moisture content between 79 and 93%, a protein content between 0.76 and 19.61%, and a fat content between 0.04 and 3.81% [11]. Rich in vitamins, phenolic compounds, carotenoids, and mineral salts [12], pumpkins can have antidiabetic, antibacterial, anticancer, and anti-obesity properties [11]. Pumpkin is widely used in food products (cakes, cookies, or bread) and is also consumed in the form of soups, smoothies, or juices [13].

Pomegranate (*Punica granatum* L.) is native to Iran (about 1.1 million tons produced per year) and is cultivated in India (2.44 million tons per year) and China (1.6 million tons per year), followed by Turkey, Afghanistan, USA, Iraq, Pakistan, Syria, and Spain [14,15]. Globally, the production of this fruit increased from 3 million tons in 2014 to 3.8 million tons in 2017 [16], and it is currently estimated that a total of 6.54 million tons of pomegranates are produced worldwide per year [15]. Fresh fruits have a moisture content of 78%, a protein content of 1.6%, a total sugars content of 14.6%, and an acidity of 0.58% [17]. Additionally, pomegranate contains tannins, anthocyanins, phenolic (gallic acid, ellagic acid), and organic acids (malic acid) which may contribute to health-promoting effects such as protection against cardiovascular disease, diabetes, skin damage, and cancer [18,19]. Pomegranate is usually consumed fresh, but also in the form of juices, alcoholic beverages, jams, and jellies [14].

In the last years, the growing demand for healthy natural foods has led to an increase in the consumption of fresh fruit juices [20], which are rich in vitamins, minerals, pigments, antioxidants, and bioactive compounds [21,22]. Fresh fruit juices have a relatively short shelf-life which can be extended by using different thermal (conventional, microwave heating, ohmic heating, and infrared radiation) and non-thermal (high-pressure processing, pulsed electric fields, ultraviolet radiation, and sonication treatments) processing methods [21,22,23,24]. However, the nutritional compounds and organoleptic characteristics of fruit juices can be affected by preservation technologies [22].

Herbs and spices have been used since ancient times to prevent food from spoiling and to enhance its sensory qualities [25,26].

Garlic (*Allium sativum* L.) is a spice used as food or medicine [27] that contains flavonoids, organosulfur, and phenolic compounds which are responsible for its biological activities [28,29]: antioxidant, anti-inflammatory, antimicrobial, and immuno-modulatory [30]. According to Lidiková et al. [31] and Melguizo-Rodríguez et al. [30], fresh garlic contains 65% water, 28% carbohydrates, 2.3% organosulfur compounds, 2% protein, 1.2% free amino acids, and 1.5% fiber. Garlic cloves are cut, dried, and ground/pulverized to obtain garlic powder, which contains organosulfur compounds such as Alliin and γ-Glutamyl-L-cysteine peptides and different percentages of allicin and its derivatives [32,33]. In food products, garlic can be used as a natural preservative, inhibiting many microorganisms such as *Bacillus cereus*, *Staphylococcus aureus*, *Lactobacillus plantarum*, *Escherichia coli*, *Salmonella typhi*, *Bacillus subtilis*, *Pseudomonas pyocyaneus*, *Candida albicans*, and a variety of yeasts [32]. The effects of garlic used for seasoning also for preserving different food products were evaluated, such as rabbit meat burgers [34], chicken sausage [35], minced beef meat [36], ready-to-eat pork patties [37], yogurt [38], soft cheese [39], and apple juice [40].

Ginger (*Zingiber officinale Roscoe*) is used as a spice [41] or seasoning for food and beverage [42] and contains phenolic (gingerols, shogaols, and paradols) and terpene compounds, polysaccharides, lipids, organic acids, and raw fibers [43]. Fresh ginger contains between 85 and 95% of water; dehydration microbial growth is inhibited, and a new product is obtained. The dried ginger can be obtained by hot air drying, freeze drying, microwave drying, and infrared radiation [42]. According to An et al. [42], the total phenolic in fresh ginger is 11.97 mg GAE/g d.w., while in dried ginger, it is between 8.41 and 13.83 mg GAE/g d.w. depending on the drying method. Phenolic compounds of ginger are inhibitors of arachidonic acid metabolism [44]. Ginger has an antimicrobial effect on various microorganisms (*S. typhi*, *E. coli*, and *B. subtilis*), and gingerol as well as shagelol are considered the more active agents [45] which lead to swelling and rupture of the bacterial cell [46]. The effects of ginger used for seasoning also for preserving different food products were evaluated, such as citrus juice [47], watermelon juice mixed with bitter gourd [48], grain syrup [49], and apple juice [40]. Regu et al. [50] investigated the effect of both garlic and ginger on the chemical, microbial, and sensory properties of cottage cheese. Moreover, the preservative effect of garlic–ginger was evaluated on cashew apple juice by Olaniran et al. [51], on fresh apple juice by Ekanem and Ekanem [52], on apple juice by Okokon and Okokon [40], and on cashew, pineapple, and watermelon juice by Olaniran et al. [53].

This study aimed to investigate the effects of natural preservatives on the physicochemical (water activity, active acidity, titratable acidity, total soluble solids contents, vitamin C, electrical conductivity, and color parameters) and microbiological characteristics of fruit juices during storage at room and refrigeration temperatures. Garlic and ginger powders were the natural preservatives added to the fruit juices (apple, apple and pumpkin, and apple and pomegranate) considered in this research. The physicochemical and microbiological characteristics of all samples were determined immediately after their preparation and during storage (days 3, 6, and 9).

## 2. Materials and Methods

### 2.1. Materials and Reagents

Fruit juices were produced in the faculty laboratory from fruits (Jonathan apple, pumpkin, and pomegranate) purchased from local supermarkets. Preservatives, namely garlic and ginger powders, were prepared in the laboratory. Ginger rhizomes and garlic cloves were purchased from a local market in Suceava, Romania. They were previously washed under flowing tap water, peeled, and diced into cubes of approximately 100 g separately. The cubes were air dried in large trays for 3 days, oven dried at 60 °C for 6 h, and pulverized with a mechanical grinding machine into powder. All chemicals and reagents (sodium hydroxide, NaOH; phenolphthalein; acetic acid; norite; diphenylhydrazine; thiourea; and sulfuric acid, H_2_SO_4_) used in the physicochemical experiments in the present study were purchased from Sigma-Aldrich (Sigma-Aldrich Chemie GmbH, Taufkirchen, Germany). Nutrient medium (nutrient agar, and malt extract agar), Petrifilm 3M Rapid *E. coli*/Coliform Count Plate, Enterobacteriaceae Count Plate, and Listeria Count Plate were purchased from Merck, Darmstadt, Germany.

### 2.2. Fruit Juice Preparation

**Figure 1:**
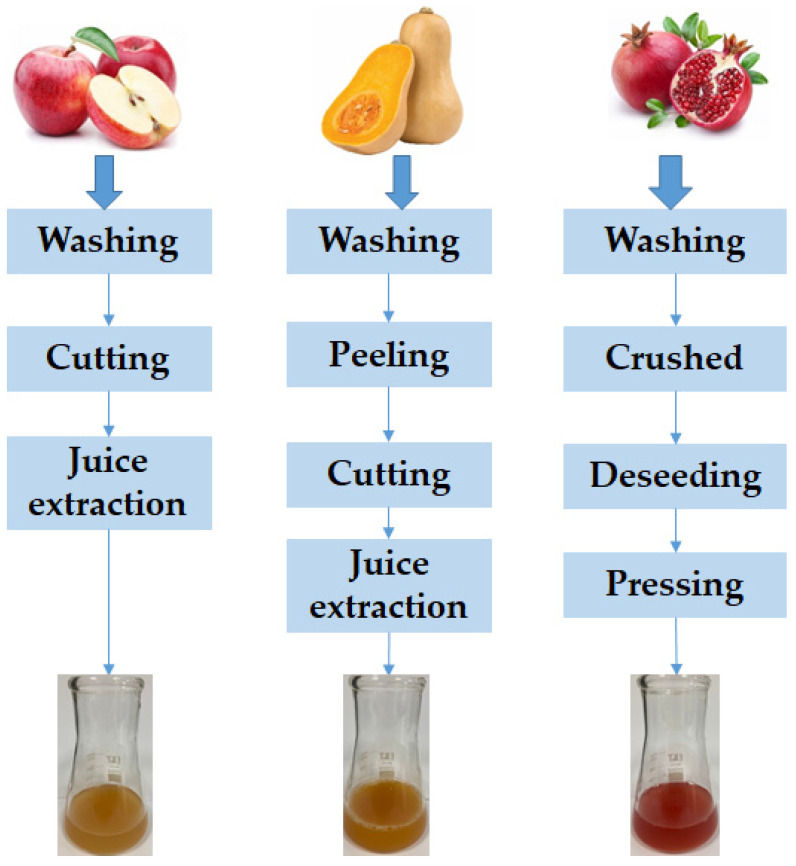
Flow diagram of processing operations for fruit juice production.

Apple, pumpkin, and pomegranate fruits were washed with tap water. The apples were cut into smaller pieces and the apple juice was extracted by cold pressing. The pumpkin was peeled, cut into large pieces, the seeds were removed, and then cut into smaller pieces from which the juice was extracted. After washing, the pomegranate fruits were crushed, peeled, and the seeds were pressed (Figure 1).

The formulation of analyzed juice assortments used in this study is presented in Table 1. Apple juice is found in all samples: 100% in samples S1, S2, S3, and S4, and 67% in the other juice samples, while pumpkin and pomegranate juices are found in a percentage of 33% in samples S5, S6, S7, and S8 and S9, S10, S11, and S12, respectively. The yield of juice extracted from pumpkin or pomegranate is small as compared with apples, and due to this fact, apple juice was used as a base for the other two formulations. The new assortments have the flavor of the added component. Samples S1, S5, and S9 are standard juice samples without commercial preservatives (untreated samples) and they were used as controls for each juice assortment. Natural preservatives such as garlic powder (0.5 g/100 mL) were added in samples S2, S6, and S10, while ginger powder (0.5 g/100 mL) was added in samples S3, S7, and S11. A combination of the two garlic (0.25 g/100 mL) and ginger (0.25 g/100 mL) powders was added in samples S4, S8, and S12. All juices were kept in bottles with airtight stoppers. The amounts of natural preservatives were added according to Ekanem and Ekanem [52].

The fruit juice samples were stored at room temperature (20 °C) and refrigeration temperature (4 °C) for 9 days. During storage, the effects of preservation with the garlic and ginger powders on the physicochemical characteristics and the microbial population of the fruit juices were monitored.

### 2.3. Physicochemical Analysis

Water activity (aw) was measured by placing an appropriate volume of apple juice sample in a special cuvette and introduced in the water activity meter AquaLab Lite (Decagon, WA, USA) chamber. The results were displayed on the device screen [4,54].

Active acidity was determined by pH measurements using a pH meter Fisher Scientific ACCUMET Bio Set AE150 (Fisher, Waltham, MA, USA) after calibration. The pH electrode was inserted into a volume of 10 mL of juice distributed in a beaker and the results were displayed on the pH meter screen [4,54].

Titratable acidity (TA) means the content of organic and mineral acids, which can be determined by titration according to the standard SR EN 12147/1999 [55]. An amount of 5 mL of juice was introduced into a 250 mL volumetric flask using a pipette and made up to the mark with distilled water, shaking well. A total of 50 mL of liquid was taken from the volumetric flask and placed in an Erlenmeyer beaker and titrated with 0.1 N NaOH in the presence of phenolphthalein as an indicator until a pale pink color appeared, persisting for 1 min. Three parallel determinations were made from the same sample. The results were expressed as % malic acid, according to other studies [54,56,57,58]. The calculation of TA values was performed based on the equation provided by Sadler and Murphy [59].

Total soluble solids contents (TSS) expressed in degrees Brix are read directly on the scale of the refractometer. The determinations are made by placing two drops of juice on the lenses of the refractometer, after prior calibration with distilled water [4,54,60].

The electrical conductivity (EC) of juice samples was measured by using a laboratory conductometer VioLab COND 51 + Set (ROTH, Karlsruhe, Germany) after calibration. The conductometer electrode was inserted into a volume of 10 mL of juice distributed in a beaker and the results were displayed on the device screen [4,54].

Vitamin C was determined by the spectrophotometric method with 2,4-dinitrophenylhydrazine as follows. The amount of sample to be analyzed was taken and quantitatively added to a 100 mL volumetric flask, then brought to the mark with acetic acid. The flask was shaken and left to rest for one hour. It was then filtered and 25 mL was taken from the filtrate, over which 0.5 g of norite was added, after which it was left at room temperature for 25 min to allow the oxidation of ascorbic acid to dehydroascorbic acid. After filtration, 4 mL of the filtrate was placed in a test tube with a rolled stopper, over which 1 mL of diphenylhydrazine and thiourea reagents in sulfuric acid were added by continuous stirring. The samples were cooled in an ice bath and 5 mL of sulfuric acid was added slowly over a period of 1 min; they were left in the dark for 30 min [3]. The samples were dosed in a HPLC SHMADZU system coupled with UV–VIS detector (DAD) using a ZORBAX-C18 column (5 μm, 250 × 4.6) (Shimadzu, Kyoto, Japan).

Color analysis was performed by using a Konika Minolta CR 400 colorimeter (Konica Minolta, Tokyo, Japan). The juice samples were introduced in a 20 mm cuvette and the following color parameters were determined: L* (whiteness/darkness), from 0 = black to 100 = white), a* (redness/greenness; −a = green, +a = red), b* (yellowness/blueness; −b = blue, +b = yellow) [4,60]. Based on the equation provided by Ghinea et al. [4], the browning index (BI) was also determined.

### 2.4. Microbiological Analysis

The total number of germs (NTG) was determined according to the standard SR EN ISO 4833-2/2014 [61]. Thus, 1 mL sample was taken from each sample and 7 dilutions were made on days 0, 3, and 6, and on day 9, 8 dilutions were made. From the last dilution, 1 mL was taken and inoculated on a Petri plate, using nutrient agar as a nutrient medium. The plates were inoculated at 35 °C for 24 h, and the colonies were counted with Funke Gerber ColonyStar (Funke Gerber, Berlin, Germany) [62].

Listeria determinations were performed according to SR EN ISO 11290-1/2017 [63]. Thus, 1 mL of sample was taken from the dilutions, inoculated on Petrifilm 3M Listeria Plate, and then incubated at 35 °C for 28 h. Colony counting was performed with Funke Gerber ColonyStar (Funke Gerber, Berlin, Germany) [64].

The numbers of Enterobacteriaceae and *E. coli* were determined according to ISO 21528-2/2017 [65] and ISO 4832/2006 [66], respectively. From the dilutions made, 1 mL of sample was taken and inoculated on Petrifilm 3M Rapid *E. coli*/Coliform Count Plate and Petrifilm 3M Enterobacteriaceae, and then incubated at 37 °C for 24 h [67,68].

Total number of yeasts and molds was determined indirectly, based on the colonies that are generated by the cells of the microorganisms present in the samples analyzed, which are formed when the sample or a dilution thereof comes into contact with a nutrient medium. Thus, 1 mL of the sample from the last dilution was taken and poured into the Petri dish containing the malt extract agar as a nutrient medium. After solidification of the medium, the plates were thermostated with the lid down at 25 °C for 72 h [67].

### 2.5. Statistical Analysis

All experiments were carried out in triplicate and the results were expressed as mean values ± standard mean error. The statistical software (Minitab version 17) was used for the statistical analysis; thus, an analysis of variance (ANOVA) with a confidence interval of 95% (*p* < 0.05) was considered for the comparison of the physicochemical and microbiological results, with the Tukey test. Additionally, principal component analysis was performed.

## 3. Results and Discussion

### 3.1. Physicochemical Analysis

**Figure 2:**
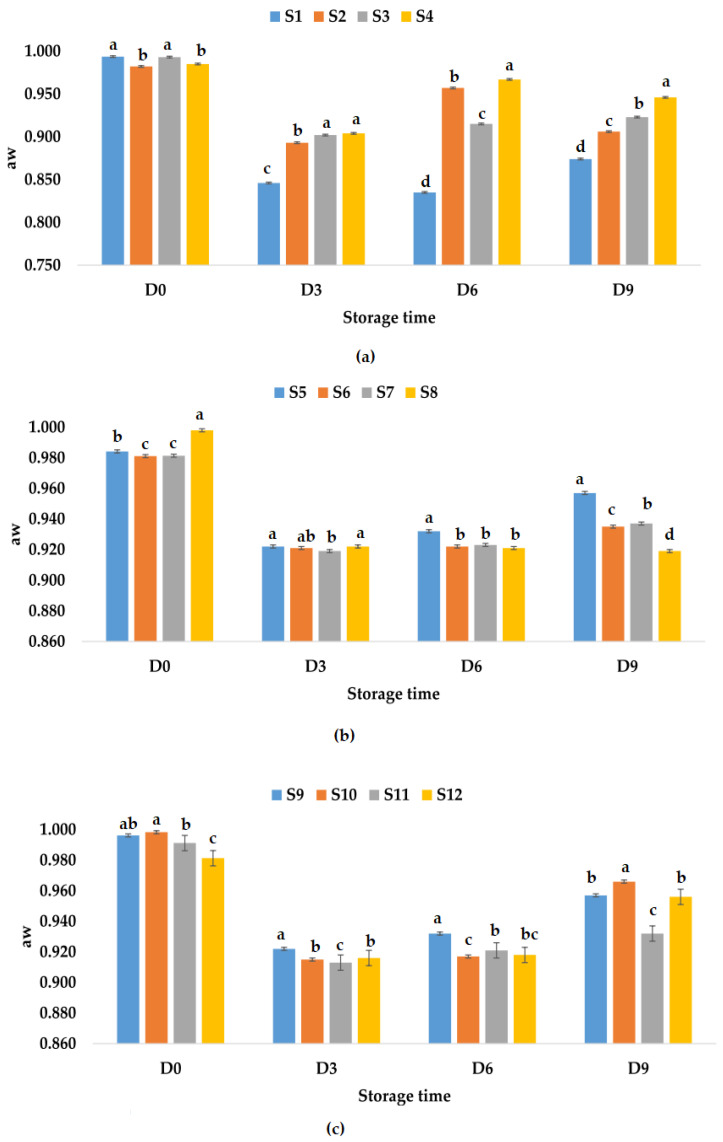
Variation in aw of juice samples during the storage period (D-day) at room temperature (20 °C): (**a**) apple juice samples (S1, S2, S3, S4); (**b**) apple and pumpkin juice samples (S5, S6, S7, S8); (**c**) apple and pomegranate juice samples (S9, S10, S11, S12). Means with different lowercase letter indicate the significant differences (*p* < 0.05) among the samples and were performed separately for each day of storage.

Water activity provides important information about the quality of a product [69], is related with most degradation reactions, and is a determinant for the growth of microorganisms according to Maltini et al. [70]. Depending on the water content, foods can be classified into three categories: low moisture (a_w_ < 0.60), intermediate moisture (a_w_ 0.60÷0.85), and high moisture (a_w_ > 0.85). Fruit juices are high moisture products, and the chances of spoilage bacteria are higher [69,70]. Water activity of apple juice samples immediately after preparation had values between 0.982 (S2) and 0.994 (S1), for apple and pumpkin juice samples (S5–S8), the values were between 0.981 (S6) and 0.998 (S8), while for apple and pomegranate juice samples (S9–S12), aw values were between 0.981 (S12) and 0.998 (S10). It was observed that samples S1 (control sample) and S3 (apple juice and ginger) had close values for aw, as well as samples S2 (apple juice and garlic) and S4 (apple juice with garlic and ginger) (Figure 2a). The aw values for the samples kept at room temperature vary over time, with a greater decrease being observed on the third day. On the ninth day, it was observed that the highest aw value was recorded for sample S4—apple juice with garlic and ginger (0.946), followed by samples S3, S2, and S1. From Figure 2b, a decrease in the aw values is observed during storage at room temperature, and on the ninth day of monitoring, the samples could be ranked in the following descending order of aw values as follows: S5 > S7 > S6 > S8. From Figure 2c, it can be observed that the values decreased until the sixth day and increased slightly on the ninth day between 0.932 (S11) and 0966 (S10).

**Figure 3:**
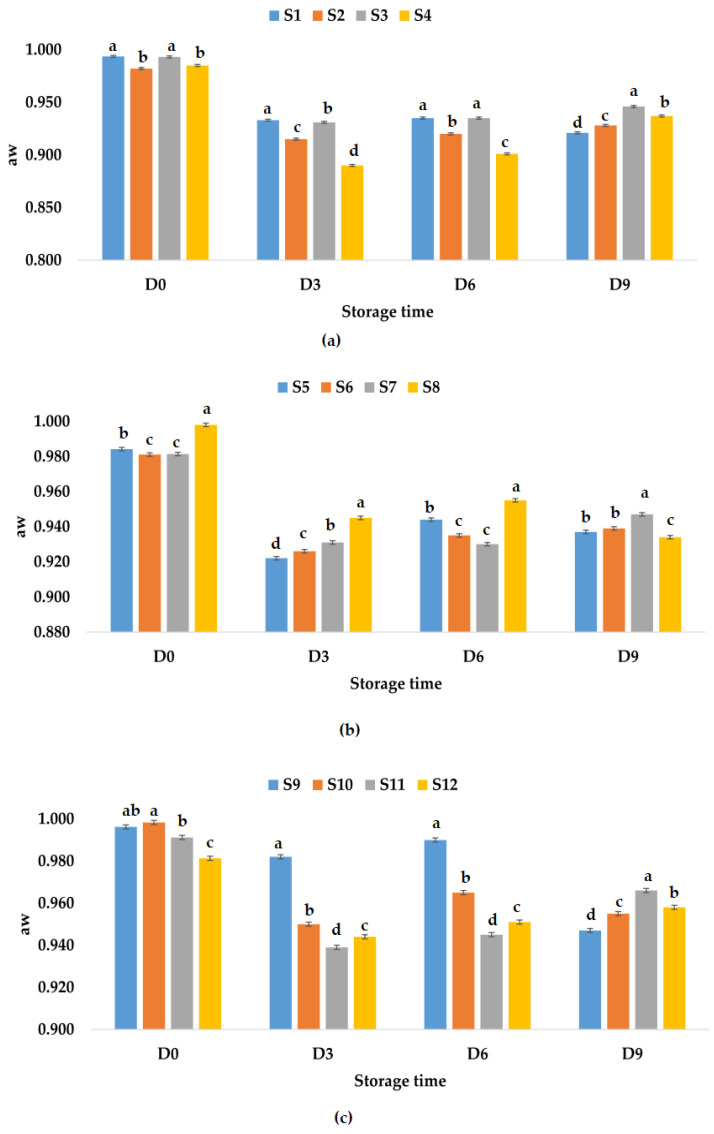
Variation in aw of juice samples during storage period (D-day) at refrigeration temperature (4 °C): (**a**) apple juice samples (S1, S2, S3, S4); (**b**) apple and pumpkin juice samples (S5, S6, S7, S8); (**c**) apple and pomegranate juice samples (S9, S10, S11, S12). Means with different lowercase letter indicate the significant differences (*p* < 0.05) among the samples and were performed separately for each day of storage.

According to Figure 3a, it can be observed that the aw values decreased much less for samples S1–S4 kept at refrigeration temperature compared to samples S1–S4 kept at room temperature. On the last day, the highest value for aw was recorded for sample S3 (0.946), while the lowest value was recorded for sample S1 (0.921). Figure 3b shows the aw values over time for samples S5–S8 stored at refrigeration temperature; on the ninth day, they can be ranked according to the aw values in the following descending order: S7 > S6 > S5 > S8. For samples S9–S12, the following hierarchy can be made: S11 > S12 > S10 > S9, taking into account the aw values recorded on the last day (Figure 3c). From our research, it can be noticed that in general the water activity of juices has decreased over time due to addition of garlic and ginger powder. The data from our study indicate that all treated juices would have a good shelf life as compared with the untreated samples.

The fresh apple juice had pH values between 3.87 and 3.89, values close to those obtained by Giryn et al. [71], Nadulski et al. [72], and Falguera et al. [73]. On the market, there are apple juices with pH values that vary from 3.0 to 4.5 [74]. Gil et al. [75] determined a pH value of 3.31, while Rydzak et al. [76] obtained pH values between 3.38 and 3.47. According to Kiskó and Roller [77], the pH of fresh apple juice can vary between 2.9 and 4.5, while pumpkin juice can have a pH between 5.34 [78] and 5.86 [79], and pomegranate juice between 2.8 and 3.6 [80] and even 3.9 [81]. The values of pH for fresh apple and pumpkin juice range from 4.36 to 4.47, which means that adding the pumpkin juice leads to an increase in pH values (are obtained juices with very little acidity), while adding the pomegranate juice to apple juice leads to a decrease in pH values (3.79–3.86). The pH of fresh fruit juice with garlic varied between 3.84 and 4.47, while the pH of fresh fruit juices with ginger ranged between 3.84 and 4.46, which are fairly close values. Additionally, the pH of fresh fruit juices with garlic and ginger varied from 3.86 to 4.45.

According to Table 2, the pH values decrease over time more for samples with only apple juice kept at refrigeration temperature compared to apple juices kept at room temperature.

The decrease is lower (11.8%) for sample S2 (apple juice and garlic) kept at room temperature, while for the samples kept refrigerated, the smallest decrease of 15.54% was observed for the sample of apple juice with ginger, followed by samples S4 and S2. In the case of samples S5–S8, the pH values decreased more for the samples kept at room temperature compared to the samples kept at refrigeration temperature. The decrease in pH values is between 11.66% (S9) and 16.42% (S10) for the samples kept at room temperature and between 14.58% (S11) and 15.51% (S12) for the samples kept at refrigeration temperature. During 9 days of storage, pH values were observed to decrease for all fruit juice samples. The same tendency was observed by Chandra et al. [48], Kaddumukasa et al. [62], and Sattar et al. [82]. Low pH values lead to the growth of acid-tolerant bacteria and to a decrease in storage stability [62]. The decrease in pH is caused by the increase in acidity and during storage may be due to the degradation of carbohydrates present in the mixed fruit juice by the action of microorganisms [83,84]. Fermentative microorganisms can tolerate low pH and the decrease in pH with storage can be attributed to these microorganisms according to Emelike and Obinna-Echem [85].

Depending on the pH values, the samples can be ranked according to Table 3.

It can be observed that all the juice samples that had ginger as a preservative, kept at refrigeration temperature, have the smallest changes in the pH values, which means that the acidity of the juices is changed very little if this is used as a natural preservative. For the juice samples only from apples and apples and pumpkin, it is observed that the mixture of ginger and garlic ranks second in terms of the influence on the pH values, while for the samples of apple and pomegranate juice, the addition of garlic would be second. It was observed that for the juice samples kept at room temperature, the control samples for both apple and pumpkin juices but also apple and pomegranate juices have the lowest decrease values, while for apple juices, the one with the lowest decrease is the one with garlic.

Malic acid is the predominant organic acid in apple fruits [4,54] and pumpkin [56], and the second most abundant organic acid in pomegranate fruits according to Nafees et al. [57], while in another study, Hasnaoui et al. [58] stated that malic acid was the prominent organic acid in the Tunisian pomegranates. Gil et al. [75] indicated a total acidity of 0.42% malic acid for apple juice, while Saad [81] determined for fresh pomegranate juice a TA value of 0.512%. Comparing the obtained results, it was observed that when pumpkin juice is added to apple juice, *TA* values decrease, and when pomegranate juice is added to apple juice, TA values increase. Additionally, when natural preservatives are added, TA values increase for samples with apple juice and samples containing apple and pumpkin juice, while TA values decrease for juices containing apple and pomegranate.

**Figure 4:**
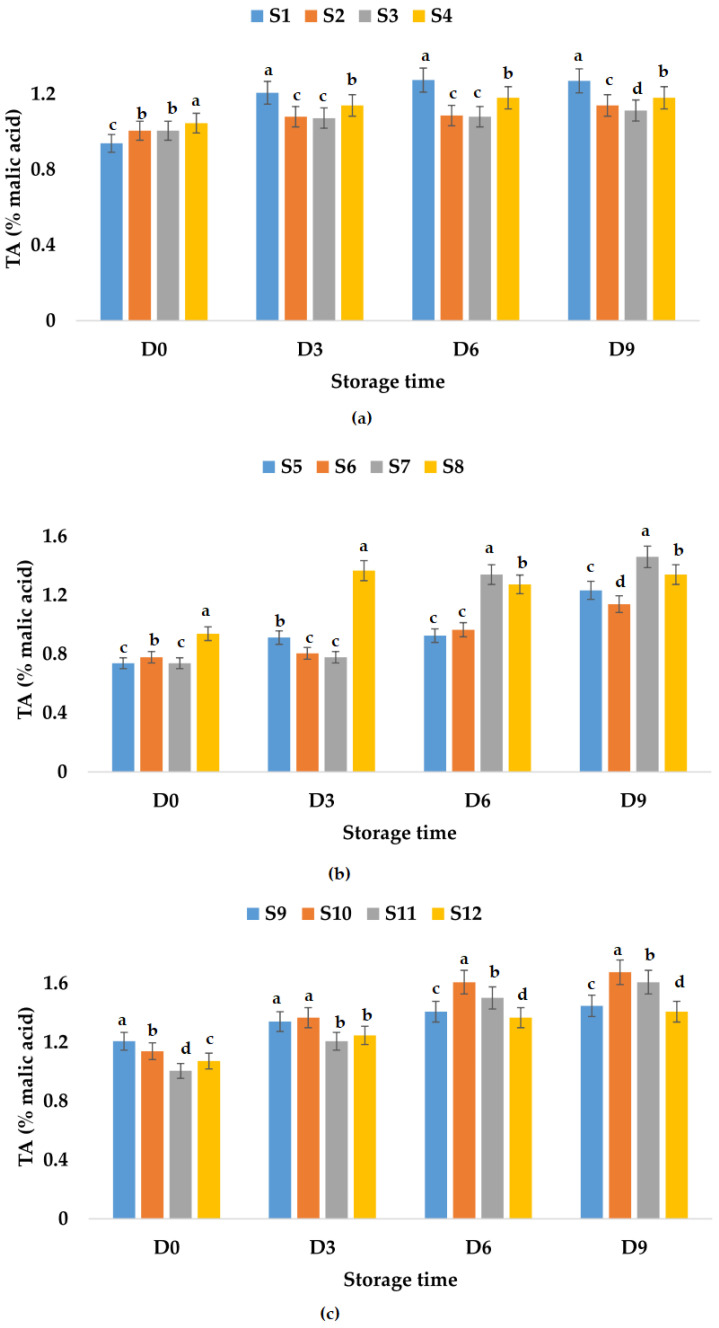
Variation in TA (% malic acid) of juice samples during the storage period (D-day) at room temperature (20 °C): (**a**) apple juice samples (S1, S2, S3, S4); (**b**) apple and pumpkin juice samples (S5, S6, S7, S8); (**c**) apple and pomegranate juice samples (S9, S10, S11, S12). Means with different lowercase letter indicate the significant differences (*p* < 0.05) among the samples and were performed separately for each day of storage.

**Figure 5:**
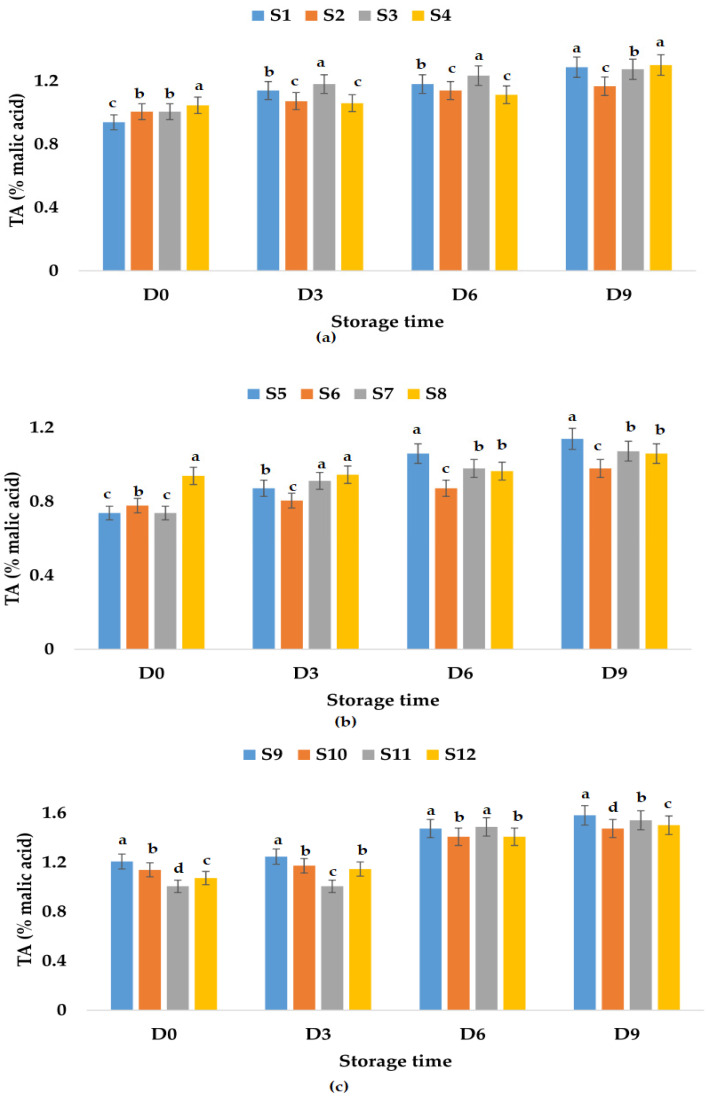
Variation in TA (% malic acid) of juice samples during the storage period (D-day) at refrigeration temperature (4 °C): (**a**) apple juice samples (S1, S2, S3, S4); (**b**) apple and pumpkin juice samples (S5, S6, S7, S8); (**c**) apple and pomegranate juice samples (S9, S10, S11, S12). Means with different lowercase letter indicate the significant differences (*p* < 0.05) among the samples and were performed separately for each day of storage.

The titratable acidity of all samples stored at room and refrigeration temperatures increased in time (Figure 4 and Figure 5). From Figure 4a, it can be observed that the highest value of TA was registered for S1, followed by S4, S2, and S3, which means that the smallest increase in the TA value (10.66%) was obtained for the apple juice sample preserved with ginger. On the other hand, in the case of apple and pumpkin juice samples, the TA values for the sample preserved with ginger showed the highest increment over time (98.18%), followed by the other samples (Figure 4b). It can be said that, in the case of this type of juice, the addition of garlic and ginger and only garlic leads to a smaller increase in TA values over time, 42.85 and 46.55%, respectively.

From Figure 4c, the TA values registered for apple and pomegranate juice samples decreased in the following order: S10 > S11 > S9 > S12, and it seems that the addition of both garlic and ginger as preservatives will lead to less increase over time in TA values (31.25%). Figure 5 shows that the addition of garlic in all the apple juice and apple juice with pomegranate fruit juice samples kept at refrigeration temperature leads to a much smaller increase in TA values compared to the control samples or the samples in which ginger or garlic and ginger were added. The TA values increased by only 12.86% for the apple and pumpkin juice sample preserved with garlic and ginger and by 25.86% for the sample preserved with garlic.

**Figure 6:**
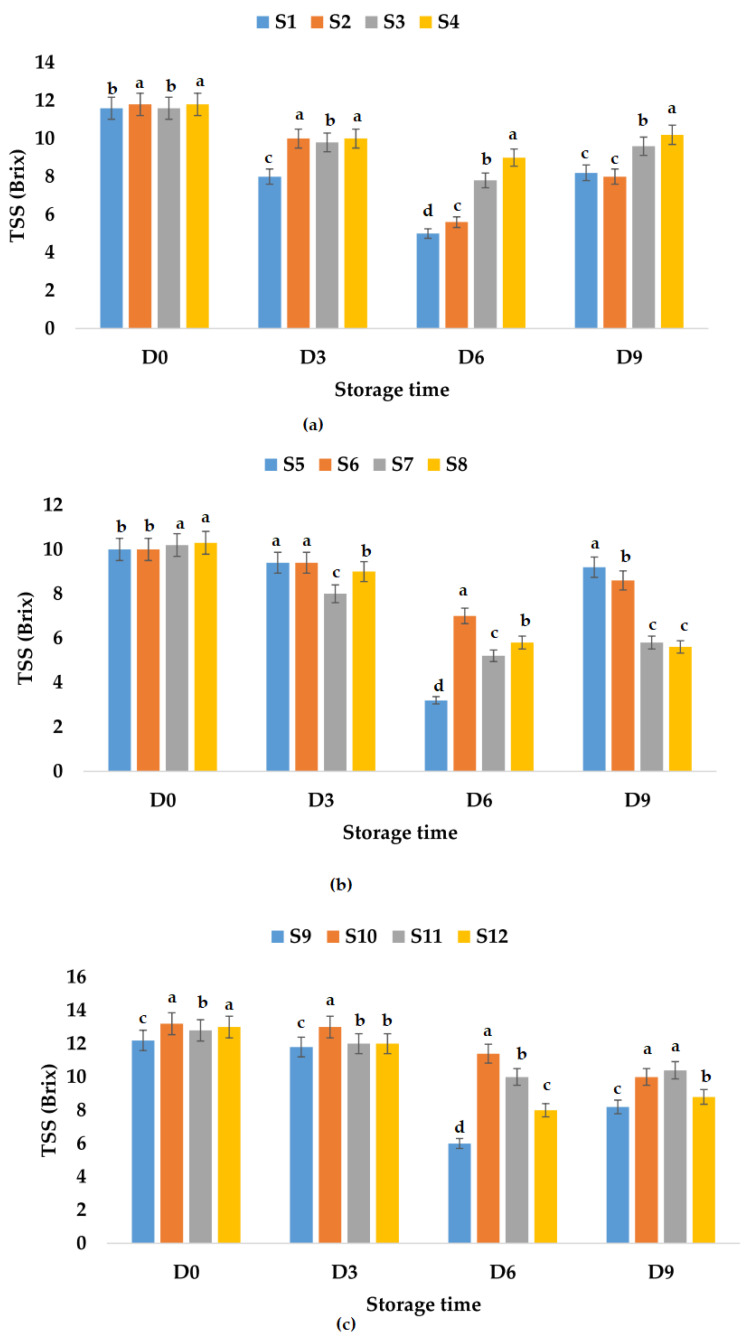
Variation in TSS (°Brix) of juice samples during the storage period (D-day) at room temperature (20 °C): (**a**) apple juice samples (S1, S2, S3, S4); (**b**) apple and pumpkin juice samples (S5, S6, S7, S8); (**c**) apple and pomegranate juice samples (S9, S10, S11, S12). Means with different lowercase letter indicate the significant differences (*p* < 0.05) among the samples and were performed separately for each day of storage.

**Figure 7:**
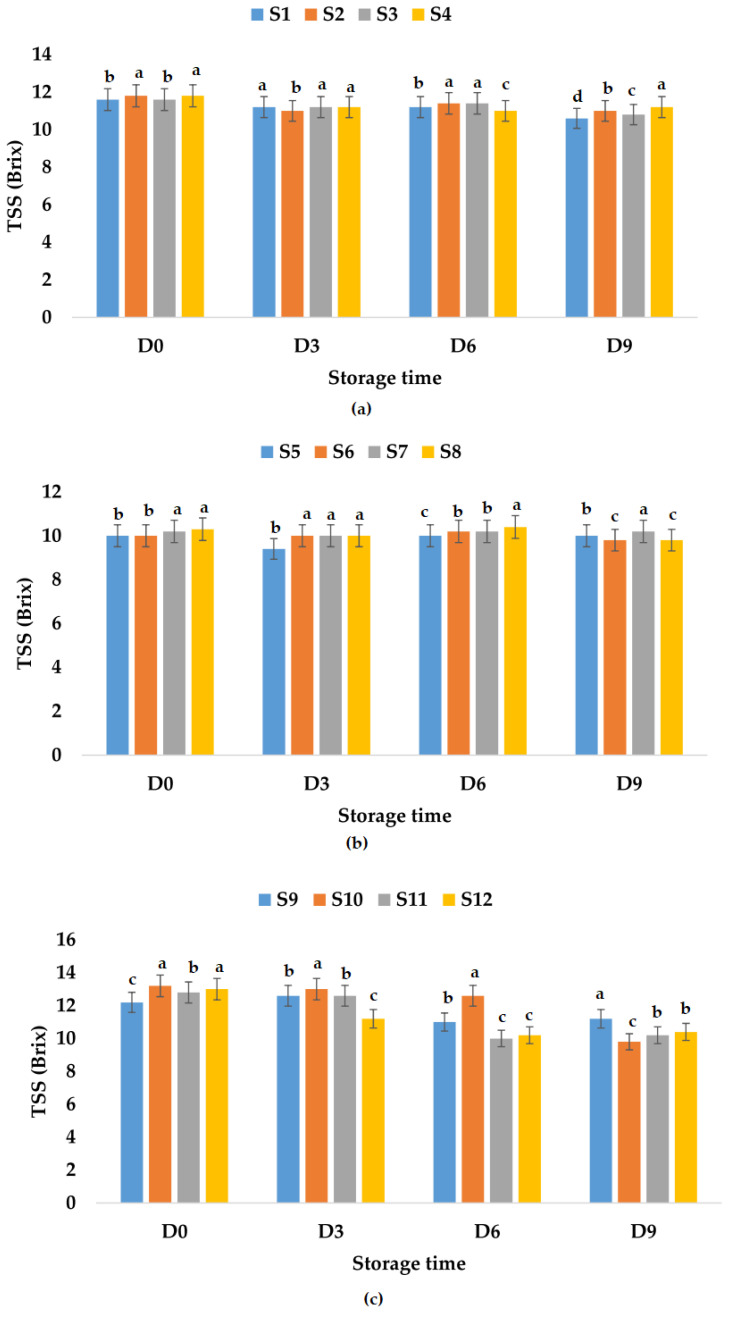
Variation in TSS (°Brix) of juice samples during the storage period (D-day) at refrigeration temperature (4 °C): (**a**) apple juice samples (S1, S2, S3, S4); (**b**) apple and pumpkin juice samples (S5, S6, S7, S8); (**c**) apple and pomegranate juice samples (S9, S10, S11, S12). Means with different lowercase letter indicate the significant differences (*p* < 0.05) among the samples and were performed separately for each day of storage.

TSS values for fresh apple juice samples ranged from 11.60 (S1, S3) to 11.80 (S2, S4) °Brix, which is within the values determined by Rydzak et al. [76] (10.77–12.07 °Brix), and were between 10.00 (S5, S6) and 10.30 (S8) °Brix for fresh apple and pumpkin juice samples. It was observed that the TSS value decreased in the case of apple and pumpkin juice due to the addition of pumpkin juice which has lower TSS values compared to apple juice (7.3 °Brix according to Allam et al. [79] or 3.1 °Brix according to Wang et al. [78]). Regarding TSS values for fresh apple and pomegranate fruit juice samples, they ranged between 12.2 (S9) and 13.0 (S12) °Brix. The results show an increase in TSS values due to the addition of pomegranate juice which according to Tarantino et al. [80] can have values between 16.2 and 17.8 °Brix. The TSS of fresh fruit juice with garlic varied between 10.00 and 13.20 °Brix, while the TSS of fresh fruit juices with ginger ranged between 10.20 and 12.80 °Brix. Additionally, the TSS of fresh fruit juices with garlic and ginger varied from 10.30 to 13.00 °Brix. It was observed that for all investigated fruit juice samples, the recorded TSS values decreased during storage (Figure 6). Figure 6 indicates the lowest value of TSS for S2, S8, and S9, leading to the conclusion that apple juice preserved with garlic would rank before apple juices preserved with ginger or with garlic and ginger, as well as S8 preserved with garlic and ginger for apple and pumpkin juices. However, taking into account the decrease over time, the ranking is slightly different. For the apple juice samples stored at room temperature, the following TSS reduction percentages were calculated: 32.20% (S2), 29.31% (S1), 17.24% (S3), and 13.55% (S4). In the case of apple and pumpkin juice stored at the same temperature, the lowest decrease in TSS over time was calculated for S5 (8.00%), followed by S6 (14.00%), while the highest was recorded for S8 (45.63%). The TSS values for apple and pomegranate juice samples stored at room temperature decreased between 18.75% (S11) and 32.79% (S9). The decrease in TSS values over time was between 0.00% (S5, S7) and 4.85% (S8) for apple and pumpkin juice stored at refrigeration temperature, and also lower in the case of apple juice samples between 5.08% (S4) and 8.62% (S1) compared with TSS values obtained for apple and pomegranate juice between 8.20% (S9) and 25.76% (S10) (Figure 7).

**Figure 8:**
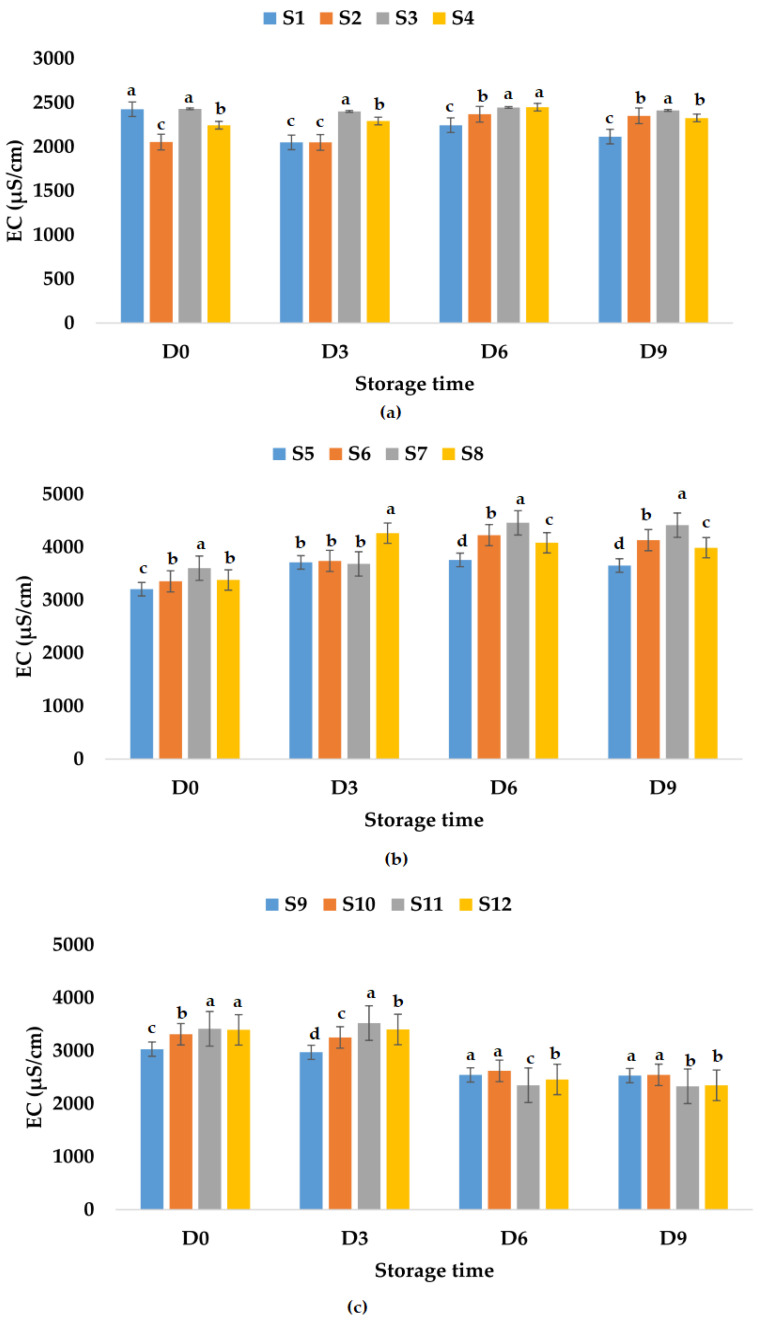
Variation in EC (μS/cm) of juice samples during the storage period (D-day) at room temperature (20 °C): (**a**) apple juice samples (S1, S2, S3, S4); (**b**) apple and pumpkin juice samples (S5, S6, S7, S8); (**c**) apple and pomegranate juice samples (S9, S10, S11, S12). Means with different lowercase letter indicate the significant differences (*p* < 0.05) among the samples and were performed separately for each day of storage.

**Figure 9:**
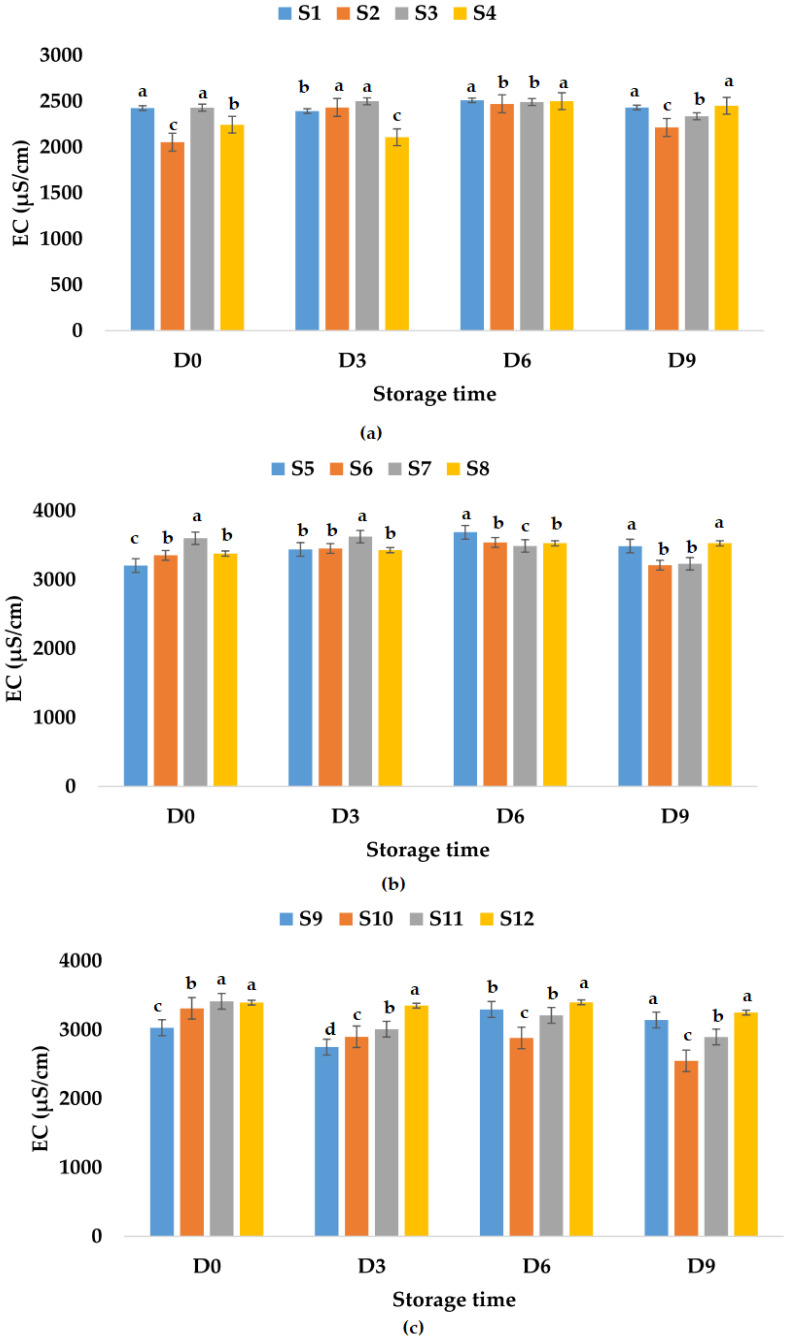
Variation in EC (μS/cm) of juice samples during the storage period (D-day) at refrigeration temperature (4 °C): (**a**) apple juice samples (S1, S2, S3, S4); (**b**) apple and pumpkin juice samples (S5, S6, S7, S8); (**c**) apple and pomegranate juice samples (S9, S10, S11, S12). Means with different lowercase letter indicate the significant differences (*p* < 0.05) among the samples and were performed separately for each day of storage.

Electrical conductivity is influenced by chemical components, ionic activity, and viscosity of liquids [86] and can be a rough measure of the relative amount of mineral substances present in the juice [87]. Among others, electrical conductivity has applications in nondestructive quality inspection in the food industry [88,89]. Palaniappan and Sastry [90] indicated that EC is influenced by the temperature and soluble solids content. EC can increase with temperature and decrease with increasing soluble solids content in fruit juices [90]. The results show that fresh fruit juices have EC values between 2425 μS/cm (apple juice) and 3205 μS/cm (apple and pumpkin juice). The EC of fresh apple and pomegranate juice was 3031 μS/cm (Figure 8). The addition of garlic decreased the EC value (2053 μS/cm) of fresh apple juice and increased the EC values of apple and pumpkin juice (3353 μS/cm) and apple and pomegranate juice (3312 μS/cm). Ginger addition in fresh fruit juices increased the EC values (2340–3602 μS/cm), while garlic and ginger addition decreased the EC value of apple juice and increased the EC values of the other two types of fruit juices (Figure 8). EC values varied in time for all analyzed samples. The highest EC value for apple juice samples stored at room temperature was determined for S3, while the lowest EC value was obtained for S1 (Figure 8a). The EC values for apple and pumpkin juice samples increased on the last day of investigation (ranged between 3653 μS/cm (S5) and 4415 μS/cm (S7), Figure 8b) compared with the EC values from the first day, while for apple and pomegranate juice samples, they decreased with values between 2330 μS/cm (S11) and 2545 μS/cm (S10) (Figure 8c). In the case of samples stored at refrigeration temperature, the EC values decreased for samples S3, S6, S7, S10, S11, and S12 and increased for the other samples on the last day of investigation (Figure 9).

**Figure 10:**
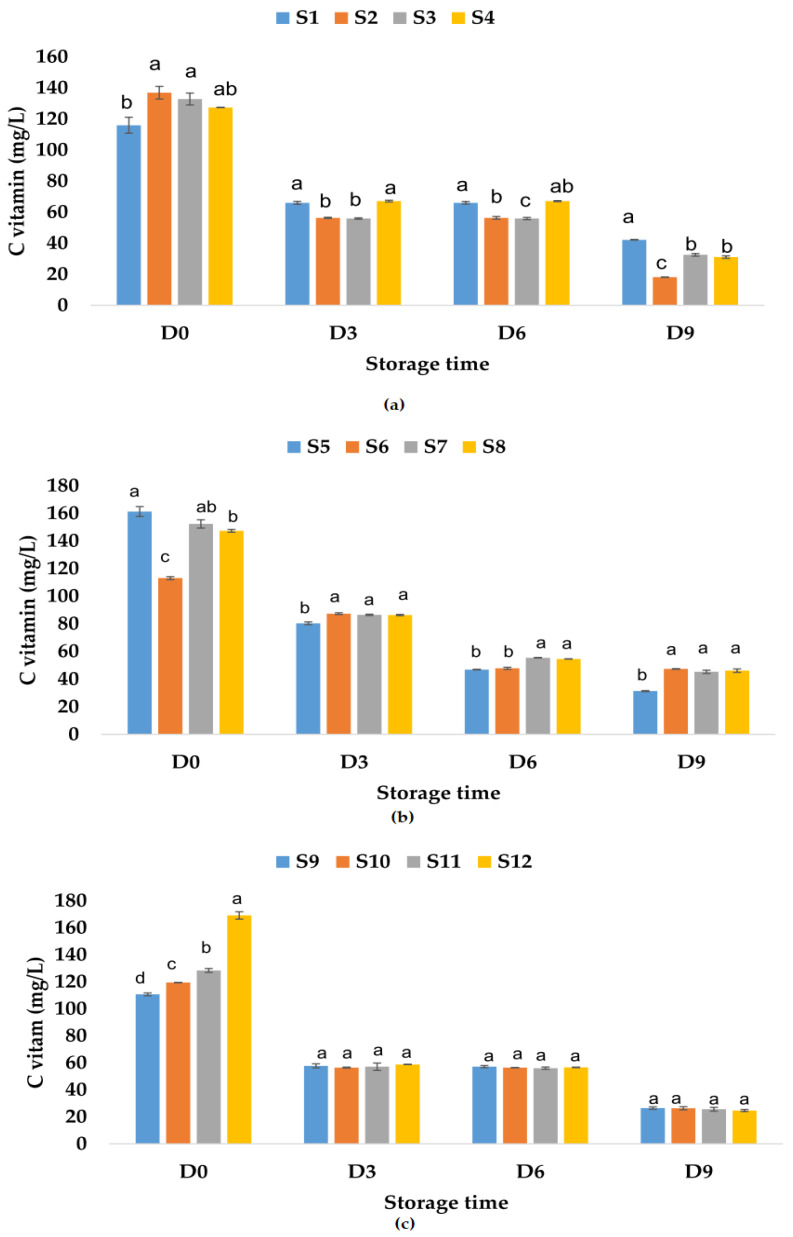
Variation in vitamin C (mg/L) of juice samples during the storage period (D-day) at room temperature (20 °C): (**a**) apple juice samples (S1, S2, S3, S4); (**b**) apple and pumpkin juice samples (S5, S6, S7, S8); (**c**) apple and pomegranate juice samples (S9, S10, S11, S12). Means with different lowercase letter indicate the significant differences (*p* < 0.05) among the samples and were performed separately for each day of storage.

The vitamin C content determined in fresh fruit juices was 115.94 mg/L of apple juice, much lower than the values reported by Falguera et al. [73] (277.4–826.6 mg/L fresh-made apple juices), 161.33 mg/L of apple and pumpkin juice, and 110.7 mg/L of apple and pomegranate juice (Figure 10).

**Figure 11:**
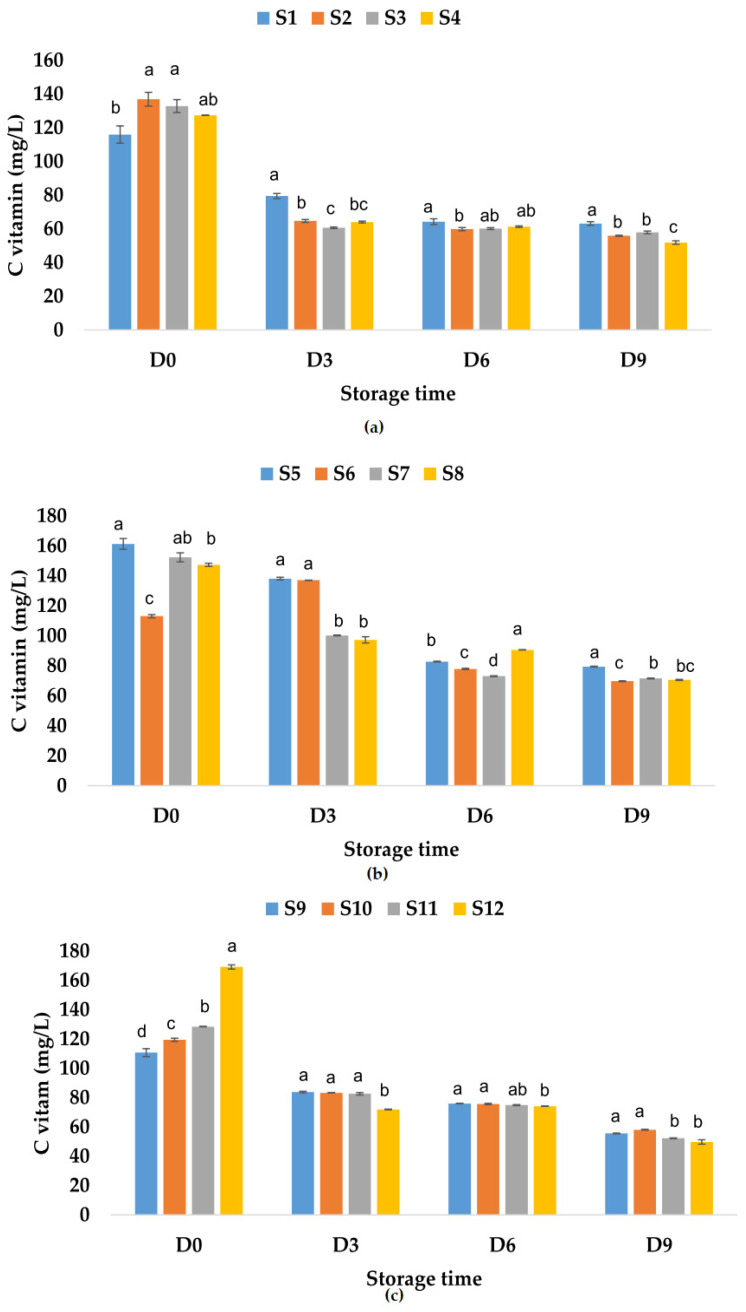
Variation in vitamin C (mg/L) of juice samples during the storage period (D-day) at refrigeration temperature (4 °C): (**a**) apple juice samples (S1, S2, S3, S4); (**b**) apple and pumpkin juice samples (S5, S6, S7, S8); (**c**) apple and pomegranate juice samples (S9, S10, S11, S12). Means with different lowercase letter indicate the significant differences (*p* < 0.05) among the samples and were performed separately for each day of storage.

These results indicate that the addition of pumpkin juice increases the vitamin C content, while the pomegranate juice (which can have a vitamin C content between 76.7 and 226.8 mg/L according to Topalović et al. [91]) added to the apple juice decreases the vitamin C content. The addition of garlic increases the vitamin C content in apple juice and apple–pomegranate juice and decreases this content in the apple–pumpkin juice sample. The same trend was observed for the samples in which only ginger or garlic and ginger were added. The highest recorded values for vitamin C content for fruit juice samples to which natural preservatives have been added are the following: 136.89 mg/L of apple juice with garlic, 152.38 mg/L of apple–pumpkin juice with ginger, and 169.14 mg/L of apple–pomegranate juice with garlic and ginger. The results showed that during storage at room temperature, the vitamin C content decreased with 63.62% for S1, 75.53% for S3, 75.61% for S4, and 86.76% for S2 (Figure 10a). In the case of apple–pumpkin juice samples, the decrease was between 58.06% (S6) and 80.56% (S5), while for apple–pomegranate juice, the decrease in vitamin C content was determined between 76.07% (S9) and 85.40% (S12). On the last day of storage, the highest vitamin C contents were recorded in the following samples: apple juice without preservatives (S1) (Figure 10a), apple–pumpkin juice with garlic (S6) (Figure 10b), and apple–pomegranate juice without preservatives (S9) and with garlic (S10) (Figure 10c). Vitamin C content also decreased during the storage of apple juice samples at refrigeration temperature by 45.53% for S1, 56.40% for S3, 59.18% for S2, and 59.27% for S4. The vitamin C content also decreased during the storage of the other juice samples as follows: for the apple–pumpkin juice samples, the calculated percentage of decrease was between 38.41% (S6) and 53.09% (S7), while for the apple–pomegranate juice samples, it was between 49.83% (S9) and 70.59% (S12). Vitamin C can be easily oxidized and decomposed during storage [92], especially in the early stage of fruit juice storage [93]. The highest values determined for vitamin C content in fruit juice samples on the last day of storage at refrigeration temperature were as follows: for apple juice–63.15 mg/L (S1), for apple–pumpkin juice–79.38 mg/L (S5), and for apple–pomegranate juice 58.09 mg/L (S10) (Figure 11).

Color is a parameter of juice quality and one of the main factors influencing consumer preferences [94,95]. Table 4 shows the color parameters of fresh fruit juices with or without natural preservatives. According to Hunterlab [95], low values of L* (0–50) indicate dark samples. The results of the present study indicate that the obtained values do not exceed 50: for the fresh apple juice, a value of 21.42 was obtained for the L* parameter, which is within the range reported by Ghinea et al. [54] (19.57–28.77). The brown or dark brown color of apple juice is due to the formation of brown pigments (melanin) during the enzymatic browning reaction, in which polyphenol oxidase oxidizes o-diphenols [86]. The obtained results showed that sample S9 is the darkest (L* = 16.47), sample S7 is the lightest (L* = 29.57), samples with pomegranate juice are the darkest, while samples containing pumpkin juice are the lightest. The addition of pomegranate juice (with L* values between 20.30 and 25.60 according to Tarantino et al. [80]) darkens the color of the apple juice, while the addition of pumpkin juice (with L* values between 52.70 and 59.48 according to [79,96]) lightens it. Based on the results obtained in the present study, it was observed that the addition of garlic to fresh fruit juices increases the L* value only for apple and pomegranate juice and decreases the L* value for the other two types of fruit juices. The same trend was observed when garlic and ginger were added to fresh fruit juices, while the addition of ginger to fresh apple juice decreased the L* value and increased it when it was added to the other two types of juices. Ginger can be chosen as anti-browning agents for apple and pumpkin or apple and pomegranate juices. Weerawardana et al. [97] determined a higher percentage of inhibitory activity of ginger on polyphenol oxidase and peroxidase enzymes, while garlic can be used to lighten the color of apple and pomegranate juice.

The samples containing only apple juice are green (negative values of the color parameter a*) and the others have positive values for the parameter a*, which means red color. Negative values for the color parameter a* were reported also by Ghinea et al. [54] between −0.96 and 0.05 for fresh apple juice, while for pomegranate juice, Tarantino et al. [80] indicated values between 4.1 and 13.00, and for pumpkin juice, Allam et al. [79] obtained a* = 6.68. The red color of pomegranate juice is due to natural monomeric anthocyanin pigments [98,99], the predominant being cyanidin-3,5-diglucoside and delphinidin-3,5-diglucoside according to Micó-Vicent et al. [100]. The results obtained in the present study indicate that the mixture of garlic and ginger used to preserve the juice samples intensifies the red color the most, while garlic decreases the value of the a* parameter, as does ginger, compared to the a* value obtained for the control sample. Adding garlic to apple and pumpkin juice increases the color intensity, as does the ginger and garlic mixture. On the other hand, adding only ginger lightens the color (more towards green) of the sample. The addition of garlic and ginger to the apple juice samples results in positive values for the color parameter a* (red color), while the addition of garlic alone enhances the green color (Table 4). For all juice samples, only positive values were obtained for the color parameter b* (yellow), the highest values were obtained for apple and pumpkin juice, and it was observed that the addition of preservatives decreases the color intensity. The yellow-orange color of pumpkin is due to the presence of carotenoids [101] such as α and β-carotene, β-cryptoxanthin, lutein, and zeaxanthin [102,103].

The color parameters of the fruit juices were also investigated during storage at room temperature and at refrigeration temperature; the obtained results are illustrated in Table 5. The L* values varied during the storage of the juices at room temperature. It was observed that for the juice samples S4, S5, and S7, the L* values decreased compared to those obtained for the fresh juices, indicating that the color between these juices darkened during storage, while for the other juice samples, their color lightened over time. The highest changes of L* were observed in apple juice with pomegranate samples S10 (64.75% increase), S11 (58.11%), and S12 (44.81%), but also in apple juice with pumpkin and garlic added (55.87%). In the case of fruit juices stored at 4 °C, the values recorded for the color parameter L* decreased on the last day of storage compared to the values on the first day for samples S5 (with 8.95%), S7 (24.59%), S9 (13.48%), S10 (25.04%), and S11 (21.89%), which means that the color of these samples darkened, while for the other analyzed samples, the color became lighter.

The influence of temperature on the color parameter L* is best observed in the case of apple and pomegranate juice samples. The pomegranate juice color is very easily affected by storage conditions [104]. The temperature at which fruit juices are stored influences the color parameter a*, especially in the case of apple juice samples (S1–S4), followed by apple and pumpkin juice samples. The storage of fruit juices at room temperature influences the color parameter b* a lot in the case of apple and pomegranate juice samples, and the least influence is observed in the case of apple and pumpkin juices with garlic and ginger, but also in the case of apple juices with ginger. The variation of the b* parameter values is smaller in the case of juice samples stored at refrigeration temperature compared to those stored at room temperature, and the greatest changes were observed for apple and pomegranate juice samples S11 (with a decrease of 64.64%) and S12 (with a decrease of 55.56%). The color alteration of red juices is influenced by non-enzymatic browning, anthocyanins degradation, and polymerization [105]. Browning index determination is a common quality control method for juice treatment conditions and loss of juice nutritional value according to Aghajanzadeh et al. [24]. BI depends on processing time and temperature [98]. The highest browning index was observed in untreated fresh fruit juices (Table 4), and after addition of natural preservatives, BI decreased. Xu et al. [106] indicated the same trend with BI fresh apple juices and after treatment with individual cinnamon essential oil. During storage, the color changing is due to enzymatic browning and occurrence of browning can be indicated by a decrease in L* value and an increase in a* or b* value [107]. The BI varied during storage; thus, for the samples stored at room temperature, namely S1, S5, and S11, a decrease in the BI values was observed compared to those obtained on the first day, and for the other samples, the BI values increased. The highest increase was observed for sample S4 (apple juice with garlic and ginger, increase by 72.27%), followed by sample S7 with an increase in BI value of 68.13%. In the case of the samples stored at refrigeration temperature, on the last day, a decrease in the BI values was observed for most of the samples with the exception of S7, S9, and S10 for which the BI values increased by 55.57%, 17.78%, and 54.97%, respectively, compared to the values obtained on the first day.

### 3.2. Microbiological Analysis

The microbiological characteristics of fruit juice samples were determined after production and during storage. Total number of yeasts and molds (TYM) per medium malt extract agar, total number of aerobic mesophilic germs per medium nutrient agar (TAMG), *E. coli*, Enterobacteriaceae, and Listeria were calculated (Table 6 and Table 7). The only safety criterion specified in the Regulation of European Commission (EC) No 2073/2005 of 15 November 2005 [108] for unpasteurized fruit and vegetable juices (ready-to-eat) is the limit of *E. coli* bacteria minim 100 CFU/g and maxim 1000 CFU/g and the absence of *Salmonella*. In this study, for all fruit juice samples, the *E. coli*, Enterobacteriaceae, and Listeria was < 1·10^4^ CFU/cm^3^. Initial counts of the total number of aerobic mesophilic germs were between 7.36 and 7.71 log_10_ CFU/cm^3^, while for total yeast and mold, counts ranged from 5.52 to 6.54 log_10_ CFU/cm^3^ (Table 6).

Starek et al. [109] stated that the total number of microorganisms in fruit juices can range from 2 to 7 log_10_ CFU/cm^3^. The microbial counts of fresh fruit juices determined in this study were within the range 2.98 to 7.64 log_10_ CFU/cm^3^ for mesophilic microorganisms (except sample S8 with 7.71 log_10_ CFU/cm^3^) and 3.79 to 6.99 log_10_ CFU/cm^3^ for yeast and molds, reported by Arı et al. [110], Dziadek et al. [111], Mosca et al. [112], and Varela-Santos et al. [113], respectively. The number of TAMG increased after the third day of storage to values between 8.11 log_10_ CFU/cm^3^ (sample S3) and 8.78 log_10_ CFU/cm^3^ (sample S6) for refrigerated samples and between 8.58 log_10_ CFU/cm^3^ (sample S4) and 9.17 log_10_ CFU/cm^3^ (sample S6) for samples stored at room temperature (Table 7).

The increase in the number of TAMG after the third day was lower (between 9.59 and 17.14%) for the refrigerated samples compared to the number of TAMG for the samples kept at room temperature (between 13.72 and 21.13%). The increase in TAMG counts on the sixth and ninth days of storage was also much higher than the initial TAMG count for samples stored at room temperature (between 21.02 and 31.36% for samples S4 and S3 and between 38.54 and 48.31% for samples S7 and S3). In this case, the results showed that the increase in TAMG number is lower in apple and pumpkin juice when ginger is added (sample S7) or in apple juice when garlic is added (sample S2) if the samples are maintained at room temperature. After the ninth day of storage at refrigeration temperature, it was observed that TAMG number increased between 32.55 and 47.19% (samples S8 and S11). The lowest increase was determined for the following juice samples: apple and pumpkin juice with garlic and ginger and apple juice with garlic. The highest increase was observed for apple and pomegranate juice with ginger stored at refrigeration temperature (47.19%) and for apple juice with ginger stored at room temperature (48.31%). The number of TYM was between 7.36 log_10_ CFU/cm^3^ (sample S3, increase with 12.47%) and 8.80 log_10_ CFU/cm^3^ (sample S2, 37.44% increase) and between 7.63 log_10_ CFU/cm^3^ (sample S3, 16.66% increase) and 9.58 log_10_ CFU/cm^3^ (sample S8, 56.17% increase) after the third day of storage at refrigeration and room temperature, respectively. However, the largest TYM increases were recorded for apple juice and pumpkin with garlic, with an increase of 50.28% and 68.22% for the samples stored in the refrigerator and at room temperature, respectively. TYM multiplied to the level of 8.66 log_10_ CFU/cm^3^ and 10.01 log_10_ CFU/cm^3^ for samples S4 and S1 stored in the refrigerator and at room temperature after the sixth day of storage. The number of TYM increased after nine days of storage between 9.73 and 10.88 log_10_ CFU/cm^3^ (samples S1 and S6 stored at refrigerator temperature) and between 10.65 and 11.03 log_10_ CFU/cm^3^ (samples S5 and S7 stored at room temperature). The largest increases (over 93%) of TYM numbers were recorded for apple and pomegranate juice samples stored at refrigerator and room temperature.

Fruit juices represent an ideal substrate for the growth of yeasts (ranging from 1.0 to 6.83 log_10_ CFU/cm^3^), especially if they have a high concentration of sugar, a low water activity, and a low pH [114]. Additionally, low pH and high sugar concentration favors the growth of molds [114].

### 3.3. Principal Component Analysis

**Figure 12:**
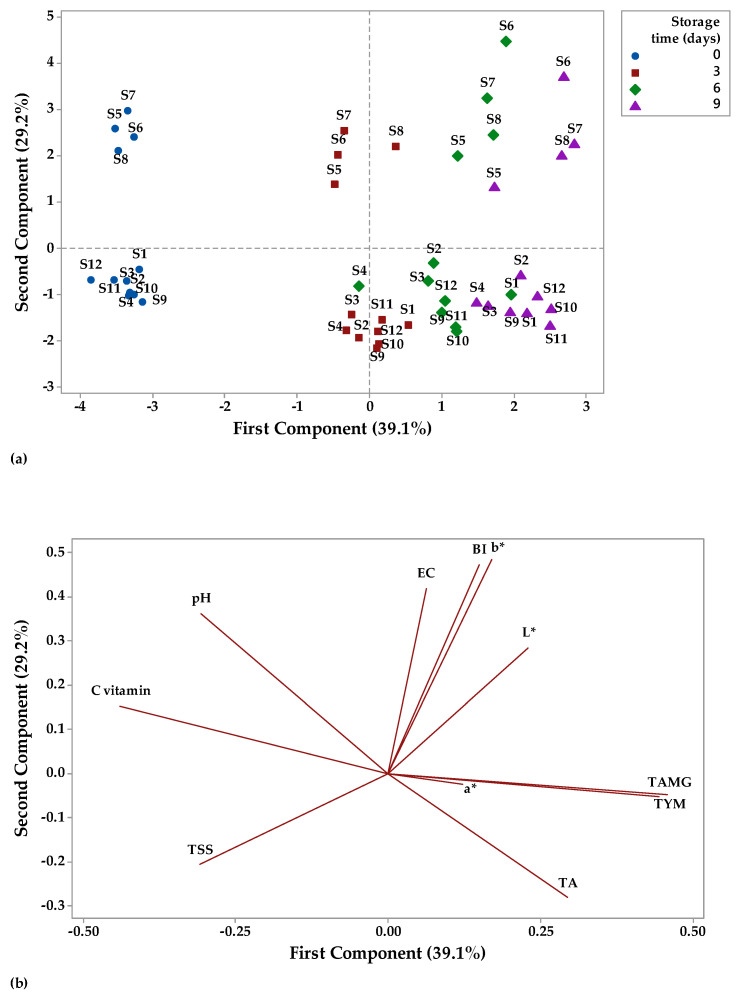
Principal component analysis (PCA) (**a**) scores and (**b**) loading plots of fruit juice samples stored at room temperature.

**Figure 13:**
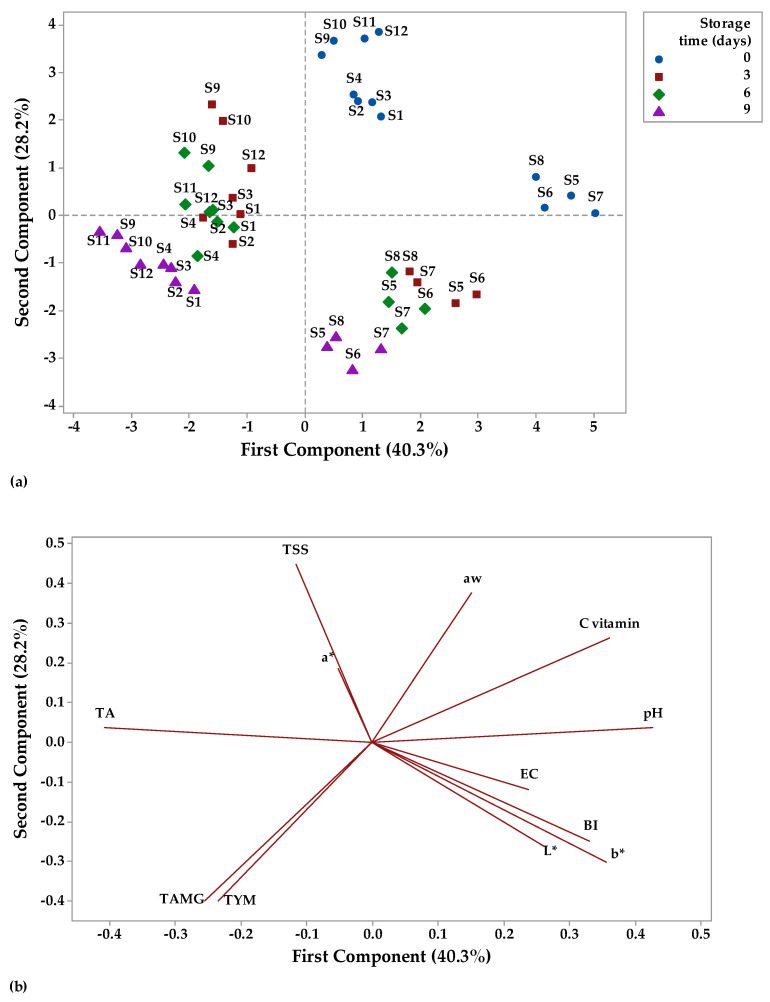
Principal component analysis (PCA) (**a**) scores and (**b**) loading plots of fruit juice samples stored at refrigerator temperature.

Principal component analysis (PCA) is a technique that can be used to classify data based on their similarities and differences [115] and to summarize a larger set of correlated variables into a smaller set [116]. In this study, the data were analyzed by PCA to highlight the relation between physicochemical and microbiological parameters of fruit juices stored at two different temperatures (Figure 12 and Figure 13). The distribution of the fruit juices according to their physicochemical and microbiological profile as affected by storage time (0, 3, 6, and 9 days) at room temperature can be visualized on a PCA scores plot (Figure 12a). A significant factor in PCA evaluation is eigenvalues higher than 1 [115]. The highest eigenvalues of 4.69 had principal component (PC1) (explained 39.1% of the total variation), while the second PC had eigenvalues of 3.50 and accounted for 29.2% of the total variation. Together, PC1 and PC2 had an account for 68.3% of the cumulative proportion of variance. Figure 12a shows that apple juice samples (S1–S4) and apple and pomegranate juice samples (S9–S12) had negative scores for PC1, while apple and pumpkin juice samples had positive scores for PC2 on day 0 of storage. For the last day of storage at room temperature, it was observed that apple juice samples (S1–S4) and apple and pomegranate juice samples (S9–S12) had positive scores for PC1, while apple and pumpkin juice samples had positive scores for PC2. The factor loadings represent the correlation between the physicochemical, microbiological parameters, and the components. Vitamin C (−0.43), pH (−0.32), aw (−0.30), and TSS (−0.28) showed negative correlation with PC1, while the other parameters were positively correlated with PC1 (the highest contribution TAMG and TYM with 0.43 each and the lowest contribution a* with 0.09). PC2 showed a negative correlation with TA (−0.24), TSS (−0.23) and TYM (−0.001), and a positive correlation with the other investigated parameters.

Overall, PC1 was mainly represented by microbiological parameters, while physicochemical parameters made larger contributions to the PC2. TAMG and TYM have large positive loadings on PC1, while vitamin C has large negative loadings on PC1. b*, BI, and EC have large positive loadings on PC2, while TSS has negative loadings on PC2 (Figure 12b).

Figure 13a illustrates the distribution of the fruit juices according to their physicochemical and microbiological profile as affected by storage time (0, 3, 6, and 9 days) at refrigerator temperature on a PCA scores plot. PC1 had the highest eigenvalues of 4.83 and explained 40.03% of the total variation, while PC2 had the eigenvalues of 3.38 and accounted for 28.2% of the total variation. Together, PC1 and PC2 had an account for 68.23% of the cumulative proportion of variance. Figure 13a indicates that all fruit juice samples had positive scores for PC2 on day 0 of storage, while on the last day of refrigerated storage, apple and pumpkin juice samples (S5–S8) had positive scores for PC1 and juice samples from apples (S1–S4); apple and pomegranate juice samples (S9–S12) had negative scores for PC1. TA (−0.40), TSS (−0.11), a* (−0.05), TAMG (−0.25), and TYM (−0.23) showed negative correlation with PC1, while the other parameters were positively correlated with PC1 (the highest contribution pH with 0.42 and the lowest contribution aw with 0.15). PC2 showed a negative correlation with TAMG (−0.39), TYM (−0.39), b* (−0.30), L* (−0.26), BI (−0.24), and EC (−0.11), and a positive correlation with the other investigated parameters (the highest contribution TSS with 0.44). Overall, PC1 was mainly represented by physicochemical parameters, while microbiological parameters made larger contributions to the PC2. pH, vitamin C, and b* have large positive loadings on PC1, while TAMG and TYM have negative loadings on PC1. TSS and aw have large positive loadings on PC2, while TAMG and TYM have large negative loadings on PC2 (Figure 13b).

## 4. Conclusions

The use of garlic and ginger as preservatives influences the physicochemical and microbiological quality of fruit juices. The stability of the physicochemical parameters of the fruit juices during nine days of storage varied depending on the type of fruit and the chosen natural preservatives. Overall, all the physicochemical parameters decreased during the storage of fruit juice samples, except TA which increased. The higher level of microbial load during the storage period was observed in all samples stored at room temperature. However, regardless of the storage temperature, the highest increases (over 93%) of the total number of yeasts and molds were recorded in the apple and pomegranate juice samples. Unpasteurized fruit juices obtained at home have a short shelf life. Natural preservation along with refrigeration can be easily done to increase the shelf life of juices. It is a simple, cheap, and convenient method, and the consumption of naturally preserved juice should be encouraged.

## Figures and Tables

**Table 1:** Formulation of analyzed juice assortments.

Samples	Fruits	Preservatives (g/100 mL)
S1	Apple (100%)	-
S2	Apple (100%)	0.5 g garlic powder
S3	Apple (100%)	0.5 g ginger powder
S4	Apple (100%)	0.25 g garlic powder + 0.25 g ginger powder
S5	Apple (67%) and pumpkin (33%)	-
S6	Apple (67%) and pumpkin (33%)	0.5 g garlic powder
S7	Apple (67%) and pumpkin (33%)	0.5 g ginger powder
S8	Apple (67%) and pumpkin (33%)	0.25 g garlic powder + 0.25 g ginger powder
S9	Apple (67%) and pomegranate (33%)	-
S10	Apple (67%) and pomegranate (33%)	0.5 g garlic powder
S11	Apple (67%) and pomegranate (33%)	0.5 g ginger powder
S12	Apple (67%) and pomegranate (33%)	0.25 g garlic powder + 0.25 g ginger powder

**Table 2:** Variation in pH of juice samples during the storage period under ambient conditions and refrigeration temperature.

Samples	D0	D3	D6	D9	% Decrease
	Stored under ambient conditions				
S1	3.88 ± 0.02 ^f^	3.46 ± 0.04 ^e^	3.44 ± 0.02 ^d^	3.40 ± 0.03 ^e^	12.37
S2	3.89 ± 0.01 ^e^	3.48 ± 0.02 ^d^	3.44 ± 0.01 ^d^	3.43 ± 0.03 ^d^	11.80
S3	3.87 ± 0.03 ^g^	3.38 ± 0.02 ^g^	3.33 ± 0.03 ^g^	3.32 ± 0.02 ^g^	14.24
S4	3.88 ± 0.01 ^f^	3.48 ± 0.03 ^d^	3.33 ± 0.02 ^g^	3.30 ± 0.01 ^i^	14.97
S5	4.36 ± 0.03 ^d^	4.01 ± 0.02 ^a^	3.61 ± 0.03 ^c^	3.60 ± 0.02 ^b^	17.44
S6	4.47 ± 0.01 ^a^	3.91 ± 0.03 ^c^	3.66 ± 0.03 ^b^	3.54 ± 0.03 ^c^	20.76
S7	4.46 ± 0.02 ^b^	3.98 ± 0.04 ^b^	3.68 ± 0.02 ^a^	3.28 ± 0.01 ^j^	26.47
S8	4.45 ± 0.03 ^c^	3.98 ± 0.03 ^b^	3.61 ± 0.02 ^c^	3.61 ± 0.03 ^a^	18.87
S9	3.79 ± 0.04 ^j^	3.44 ± 0.03 ^f^	3.35 ± 0.03 ^f^	3.35 ± 0.01 ^f^	11.66
S10	3.84 ± 0.02 ^i^	3.26 ± 0.01 ^i^	3.24 ± 0.03 ^i^	3.21 ± 0.01 ^k^	16.42
S11	3.84 ± 0.03 ^i^	3.37 ± 0.02 ^h^	3.31 ± 0.01 ^h^	3.30 ± 0.03 ^i^	14.04
S12	3.86 ± 0.01 ^h^	3.46 ± 0.03 ^e^	3.38 ± 0.01 ^e^	3.31 ± 0.03 ^h^	14.22
	Stored at refrigeration temperature				
S1	3.88 ± 0.02 ^f^	3.27 ± 0.01 ^i^	3.26 ± 0.01 ^g^	3.25 ± 0.03 ^g^	16.23
S2	3.89 ± 0.01 ^e^	3.28 ± 0.03 ^h^	3.28 ± 0.03 ^e^	3.26 ± 0.04 ^f^	16.15
S3	3.87 ± 0.03 ^g^	3.30 ± 0.02 ^f^	3.30 ± 0.03 ^d^	3.27 ± 0.01 ^e^	15.54
S4	3.88 ± 0.01 ^f^	3.29 ± 0.01 ^g^	3.28 ± 0.01 ^e^	3.26 ± 0.01 ^f^	15.99
S5	4.36 ± 0.03 ^d^	3.80 ± 0.01 ^d^	3.80 ± 0.01 ^c^	3.80 ± 0.03 ^c^	12.89
S6	4.47 ± 0.01 ^a^	3.89 ± 0.02 ^b^	3.86 ± 0.02 ^b^	3.85 ± 0.01 ^b^	13.88
S7	4.46 ± 0.02 ^b^	3.92 ± 0.01 ^a^	3.91 ± 0.02 ^a^	3.90 ± 0.02 ^a^	12.58
S8	4.45 ± 0.03 ^c^	3.87 ± 0.02 ^c^	3.86 ± 0.02 ^b^	3.85 ± 0.01 ^b^	13.51
S9	3.79 ± 0.04 ^j^	3.23 ± 0.03 ^j^	3.22 ± 0.01 ^h^	3.22 ± 0.01 ^h^	15.07
S10	3.84 ± 0.02 ^i^	3.28 ± 0.01 ^h^	3.27 ± 0.05 ^f^	3.27 ± 0.01 ^e^	14.85
S11	3.84 ± 0.03 ^i^	3.47 ± 0.01 ^e^	3.3 0 ± 0.01 ^d^	3.28 ± 0.02 ^d^	14.58
S12	3.86 ± 0.01 ^h^	3.29 ± 0.01 ^g^	3.28 ± 0.02 ^e^	3.26 ± 0.02 ^f^	15.51

Values are means and standard errors of three determinations (considering one S1–S12 data group on each day separately, and storage). Values with the different letters within one column per each day of determination are significantly different (*p* < 0.05).

**Table 3:** Ranking of juice samples according to decrease in pH values.

Type of Fruit Juice	Room Temperature	Refrigeration Temperature
Apple juice	S4 > S3 > S1 > S2	S1 > S2 > S4 > S3
Apple and pumpkin juice	S7 > S6 > S8 > S5	S6 > S8 > S5 > S7
Apple and pomegranate juice	S10 > S12 > S11 > S9	S12 > S9 > S10 > S11

**Table 4:** Color parameters of initial juice samples.

	Sample	L*	a*	b*	BI
Apple (100%) juice samples	S1	21.42 ± 0.40 ^c^	−0.14 ± 0.02 ^i^	4.55 ± 0.11 ^e^	22.81 ± 0.23 ^ef^
	S2	20.86 ± 0.24 ^c^	−0.34 ± 0.01 ^j^	4.06 ± 0.51 ^e^	19.90 ± 2.73 ^efgh^
	S3	20.49 ± 0.05 ^c^	−0.14 ± 0.03 ^i^	3.54 ± 0.03 ^f^	18.01 ± 0.24 ^gh^
	S4	20.55 ± 0.19 ^c^	0.17 ± 0.05 ^h^	3.57 ± 0.01 ^f^	19.26 ± 0.05 ^fgh^
Apple (67%) and pumpkin (33%) juice samples	S5	25.02 ± 0.48 ^b^	0.30 ± 0.02 ^g^	11.94 ± 0.02 ^a^	63.30 ± 1.42 ^a^
	S6	24.54 ± 0.97 ^b^	1.05 ± 0.02 ^e^	8.90 ± 0.02 ^c^	47.20 ± 2.11 ^b^
	S7	29.57 ± 0.20 ^a^	0.13 ± 0.04 ^h^	10.19 ± 0.75 ^b^	41.46 ± 3.47 ^c^
	S8	23.47 ± 0.95 ^b^	0.73 ± 0.03 ^f^	6.94 ± 0.17 ^d^	36.60 ± 0.65 ^d^
Apple (67%) and pomegranate (33%) juice samples	S9	16.47 ± 0.97 ^e^	3.78 ± 0.02 ^b^	1.24 ± 0.13 ^g^	23.84 ± 0.45 ^e^
	S10	18.41 ± 0.02 ^d^	3.22 ± 0.03 ^d^	0.75 ± 0.08 ^g^	16.39 ± 0.55 ^h^
	S11	17.95 ± 0.46 ^de^	3.40 ± 0.11 ^c^	1.37 ± 0.02 ^g^	21.20 ± 0.00 ^efg^
	S12	18.03 ± 0.38 ^de^	4.18 ± 0.05 ^a^	1.14 ± 0.23 ^g^	22.65 ± 1.10 ^ef^

Values are means and standard errors of three determinations. Values with the different letters within one column (all juice samples) are significantly different (*p* < 0.05).

**Table 5:** Color parameters of juice samples during storage at room and refrigeration temperatures.

	Sample	L*	a*	b*	BI
		Stored under ambient conditions			
Apple (100%) juice samples (3rd day)	S1	19.87 ± 0.05 ^c^	−0.07 ± 0.01 ^f^	4.41 ± 0.04 ^d^	24.23 ± 0.22 ^d^
	S2	20.18 ± 0.82 ^bc^	−0.15 ± 0.03 ^f^	3.32 ± 0.03 ^e^	17.01 ± 0.47 ^efg^
	S3	20.81 ± 0.01 ^b^	−0.11 ± 0.02 ^f^	4.09 ± 0.32 ^d^	20.98 ± 1.93 ^de^
	S4	22.77 ± 0.04 ^a^	−0.14 ± 0.01 ^f^	2.90 ± 0.40 ^e^	12.85 ± 1.97 ^g^
Apple (67%) and pumpkin (33%) juice samples (3rd day)	S5	22.67 ± 0.20 ^a^	0.95 ± 0.05 ^d^	7.42 ± 0.05 ^c^	41.86 ± 0.04 ^c^
	S6	22.76 ± 0.05 ^a^	0.59 ± 0.20 ^e^	9.53 ± 0.40 ^b^	54.56 ± 3.39 ^b^
	S7	23.03 ± 0.52 ^a^	1.17 ± 0.03 ^cd^	10.42 ± 0.20 ^a^	62.12 ± 0.25 ^a^
	S8	23.54 ± 0.02 ^a^	0.72 ± 0.40 ^d^	10.66 ± 0.02 ^a^	60.57 ± 1.44 ^a^
Apple (67%) and pomegranate (33%) juice samples (3rd day)	S9	17.70 ± 0.30 ^e^	1.85 ± 0.22 ^bc^	1.81 ± 0.01 ^f^	18.12 ± 0.63 ^ef^
	S10	17.65 ± 0.05 ^e^	2.17 ± 0.06 ^b^	1.92 ± 0.05 ^f^	20.16 ± 0.50 ^de^
	S11	18.74 ± 0.02 ^d^	3.00 ± 0.20 ^a^	1.81 ± 0.03 ^f^	21.41 ± 0.90 ^de^
	S12	19.71 ± 0.30 ^c^	2.23 ± 0.70 ^b^	1.44 ± 0.01 ^f^	15.51 ± 2.32 ^fg^
Apple (100%) juice samples (6th day)	S1	21.14 ± 0.90 ^g^	−0.10 ± 0.02 ^i^	5.16 ± 0.20 ^de^	26.95 ± 0.03 ^d^
	S2	24.26 ± 0.03 ^cd^	−0.17 ± 0.03 ^i^	5.65 ± 0.30 ^d^	25.35 ± 1.63 ^d^
	S3	22.91 ± 0.10 ^def^	0.65 ± 0.05 ^h^	5.23 ± 0.20 ^de^	27.46 ± 1.14 ^d^
	S4	23.65 ± 0.60 ^cde^	1.09 ± 0.01 ^g^	4.85 ± 0.84 ^def^	25.88 ± 3.69 ^d^
Apple (67%) and pumpkin (33%) juice samples (6th day)	S5	22.12 ± 0.12 ^fg^	2.02 ± 0.11 ^e^	8.57 ± 0.92 ^c^	54.77 ± 6.52 ^c^
	S6	36.15 ± 0.02 ^a^	2.54 ± 0.02 ^c^	17.90 ± 0.4 ^a^	71.16 ± 1.99 ^b^
	S7	22.38 ± 0.03 ^efg^	1.87 ± 0.05 ^f^	12.83 ± 0.50 ^b^	87.21 ± 4.55 ^a^
	S8	26.59 ± 0.50 ^b^	2.33 ± 0.02 ^d^	11.58 ± 0.40 ^b^	62.15 ± 1.07 ^c^
Apple (67%) and pomegranate (33%) juice samples (6th day)	S9	18.63 ± 0.50 ^h^	2.24 ± 0.03 ^d^	3.21 ± 0.03 ^g^	27.36 ± 0.48 ^d^
	S10	24.35 ± 0.01 ^c^	2.56 ± 0.04 ^c^	3.81 ± 0.04 ^fg^	24.37 ± 0.30 ^d^
	S11	22.41 ± 0.52 ^efg^	3.26 ± 0.03 ^a^	2.96 ± 0.02 ^g^	24.41 ± 0.40 ^d^
	S12	23.54 ± 0.80 ^cde^	3.10 ± 0.02 ^b^	3.98 ± 0.01 ^efg^	27.81 ± 0.90 ^d^
Apple (100%) Juice samples (9th day)	S1	25.73 ± 0.30 ^de^	0.06 ± 0.01 ^i^	3.96 ± 0.40 ^gh^	16.49 ± 1.62 ^i^
	S2	26.10 ± 0.90 ^d^	−0.49 ± 0.05 ^j^	6.10 ± 0.02 ^e^	24.56 ± 0.73 ^fg^
	S3	22.54 ± 0.40 ^f^	0.36 ± 0.04 ^h^	4.33 ± 0.50 ^g^	22.03 ± 2.39 ^gh^
	S4	19.65 ± 0.44 ^g^	0.63 ± 0.03 ^g^	5.31 ± 0.40 ^ef^	33.18 ± 1.97 ^d^
Apple (67%) and pumpkin (33%) juice samples (9th day)	S5	22.89 ± 0.20 ^f^	1.43 ± 0.01 ^e^	9.57 ± 0.50 ^d^	57.34 ± 2.94 ^bc^
	S6	38.25 ± 0.10 ^a^	1.09 ± 0.03 ^f^	16.84 ± 0.03 ^a^	58.29 ± 0.00 ^b^
	S7	24.34 ± 0.20 ^e^	1.77 ± 0.02 ^c^	11.83 ± 0.02 ^b^	69.71 ± 0.55 ^a^
	S8	26.79 ± 0.20 ^d^	1.63 ± 0.04 ^d^	10.58 ± 0.40 ^c^	53.50 ± 1.97 ^c^
Apple (67%) and pomegranate (33%) juice samples (9th day)	S9	19.63 ± 0.50 ^g^	2.72 ± 0.02 ^b^	3.60 ± 0.40 ^gh^	29.99 ± 1.74 ^de^
	S10	30.33 ± 0.01 ^b^	1.06 ± 0.05 ^f^	4.51 ± 0.20 ^fg^	18.33 ± 0.88 ^hi^
	S11	28.38 ± 0.60 ^c^	2.76 ± 0.02 ^b^	3.28 ± 0.30 ^h^	19.08 ± 0.82 ^hi^
	S12	26.11 ± 0.94 ^d^	3.64 ± 0.01 ^a^	4.38 ± 0.20 ^fg^	28.18 ± 0.14 ^ef^
	Stored at refrigerator temperature				
Apple (100%) juice samples (3rd day)	S1	20.93 ± 0.50 ^bc^	−0.38 ± 0.03 ^k^	3.90 ± 0.1 ^c^	18.75 ± 0.18 ^d^
	S2	21.37 ± 0.30 ^b^	−0.45 ± 0.04 ^k^	3.83 ± 0.3 ^c^	17.68 ± 1.53 ^de^
	S3	20.96 ± 0.25 ^bc^	−0.21 ± 0.02 ^j^	3.16 ± 0.6 ^cd^	15.21 ± 3.16 ^de^
	S4	19.75 ± 0.45 ^cd^	−0.08 ± 0.04 ^i^	2.38 ± 0.5 ^de^	12.22 ± 2.66 ^e^
Apple (67%) and pumpkin (33%) juice samples (3rd day)	S5	21.83 ± 0.20 ^b^	0.28 ± 0.01 ^h^	9.57 ± 0.4 ^a^	56.72 ± 2.40 ^a^
	S6	24.03 ± 0.5 ^a^	1.11 ± 0.02 ^e^	10.10 ± 0.1 ^a^	56.42 ± 0.76 ^a^
	S7	23.25 ± 0.55 ^a^	0.78 ± 0.06 ^f^	7.75 ± 0.8 ^b^	42.07 ± 3.97 ^b^
	S8	21.38 ± 0.43 ^b^	0.53 ± 0.03 ^g^	8.06 ± 0.4 ^b^	47.88 ± 1.77 ^b^
Apple (67%) and pomegranate (33%) juice samples (3rd day)	S9	17.89 ± 0.23 ^e^	2.41 ± 0.01 ^d^	1.49 ± 0.2 ^e^	18.16 ± 1.02 ^de^
	S10	17.31 ± 0.60 ^e^	3.06 ± 0.03 ^c^	2.21 ± 0.6 ^de^	26.06 ± 3.19 ^c^
	S11	19.37 ± 0.90 ^d^	3.55 ± 0.03 ^b^	1.63 ± 0.4 ^e^	21.61 ± 1.37 ^cd^
	S12	20.63 ± 0.40 ^bcd^	3.66 ± 0.04 ^a^	1.71 ± 0.2 ^e^	21.09 ± 0.79 ^cd^
Apple (100%) juice samples (6th day)	S1	22.57 ± 0.3 ^bc^	−0.23 ± 0.01 ^g^	3.98 ± 0.02 ^d^	18.17 ± 0.13 ^e^
	S2	21.32 ± 0.8 ^c^	−0.46 ± 0.04 ^h^	3.18 ± 0.6 ^de^	14.10 ± 2.78 ^ef^
	S3	22.03 ± 0.5 ^bc^	−0.32 ± 0.03 ^g^	2.81 ± 0.9 ^e^	12.23 ± 4.37 ^f^
	S4	21.40 ± 0.9 ^c^	−0.63 ± 0.03 ^i^	2.50 ± 0.4 ^e^	9.89 ± 1.72 ^f^
Apple (67%) and pumpkin (33%) juice samples (6th day)	S5	22.02 ± 0.8 ^bc^	1.01 ± 0.02 ^d^	8.82 ± 0.02 ^c^	53.28 ± 2.22 ^b^
	S6	24.95 ± 0.4 ^a^	1.22 ± 0.04 ^c^	9.82 ± 0.03 ^b^	52.37 ± 0.74 ^b^
	S7	22.96 ± 0.1 ^b^	0.22 ± 0.05 ^f^	11.05 ± 0.01 ^a^	63.80 ± 0.12 ^a^
	S8	22.65 ± 0.3 ^bc^	0.82 ± 0.02 ^e^	8.16 ± 0.04 ^c^	46.26 ± 0.42 ^c^
Apple (67%) and pomegranate (33%) juice samples (6th day)	S9	16.45 ± 0.5 ^de^	2.82 ± 0.02 ^a^	2.63 ± 0.01 ^e^	29.53 ± 0.79 ^d^
	S10	15.62 ± 0.4 ^e^	1.32 ± 0.01 ^b^	3.06 ± 0.05 ^de^	27.62 ± 0.34 ^d^
	S11	17.45 ± 0.5 ^d^	2.83 ± 0.04 ^a^	1.24 ± 0.06 ^f^	18.75 ± 0.01 ^e^
	S12	21.21 ± 0.1 ^c^	2.85 ± 0.03 ^a^	1.14 ± 0.02 ^f^	14.97 ± 0.13 ^ef^
Apple (100%) Juice samples (9th day)	S1	24.55 ± 0.9 ^ab^	−0.15 ± 0.04 ^f^	3.66 ± 0.6 ^d^	15.27 ± 2.32 ^e^
	S2	21.46 ± 0.7 ^e^	−0.36 ± 0.06 ^g^	3.68 ± 0.6 ^d^	17.06 ± 2.88 ^e^
	S3	23.63 ± 0.8 ^bcd^	−0.15 ± 0.04 ^f^	2.41 ± 0.4 ^de^	10.00 ± 1.61 ^ef^
	S4	24.40 ± 0.9 ^abc^	−0.23 ± 0.02 ^f^	2.00 ± 0.3 ^ef^	7.63 ± 1.07 ^f^
Apple (67%) and pumpkin (33%) juice samples (9th day)	S5	22.78 ± 0.4 ^bcde^	0.91 ± 0.01 ^c^	8.12 ± 0.2 ^bc^	45.98 ± 0.36 ^b^
	S6	25.67 ± 0.6 ^a^	1.00 ± 0.05 ^c^	9.09 ± 0.9 ^b^	45.54 ± 4.06 ^b^
	S7	22.27 ± 0.8 ^de^	0.10 ± 0.02 ^e^	10.85 ± 0.1 ^a^	64.50 ± 2.26 ^a^
	S8	23.95 ± 0.9 ^abcd^	0.62 ± 0.06 ^d^	7.06 ± 0.6 ^c^	36.01 ± 2.04 ^c^
Apple (67%) and pomegranate (33%) juice samples (9th day)	S9	14.25 ± 0.2 ^f^	2.12 ± 0.01 ^a^	2.30 ± 0.2 ^de^	28.08 ± 1.31 ^d^
	S10	13.80 ± 0.5 ^f^	1.82 ± 0.06 ^b^	2.06 ± 0.6 ^ef^	25.40 ± 4.44 ^d^
	S11	14.02 ± 0.2 ^f^	2.23 ± 0.03 ^a^	0.64 ± 0.4 ^f^	15.82 ± 2.93 ^e^
	S12	22.53 ± 0.7 ^cde^	2.23 ± 0.02 ^a^	0.64 ± 0.3 ^f^	9.84 ± 1.12 ^ef^

Values are means and standard errors of three determinations (considering one S1–S12 data group on each day separately, and storage). Values with the different letters within one column per each day of determination are significantly different (*p* < 0.05).

**Table 6:** Microbial counts of the fresh fruit juices (in log_10_ CFU/cm^3^).

	Sample	Total Number of Aerobic Mesophilic Germs (TAMG)	Total Number of Yeasts and Molds (TYM)
Apple (100%) juice samples	S1	7.59 ± 0.01 ^bc^	6.29 ± 0.01 ^abc^
	S2	7.64 ± 0.02 ^b^	6.40 ± 0.01 ^abc^
	S3	7.40 ± 0.03 ^gh^	6.54 ± 0.01 ^a^
	S4	7.47 ± 0.01 ^ef^	6.09 ± 0.02 ^cd^
Apple (67%) and pumpkin (33%) juice samples	S5	7.46 ± 0.03 ^fg^	6.50 ± 0.01 ^ab^
	S6	7.57 ± 0.02 ^cd^	5.67 ± 0.19 ^e^
	S7	7.47 ± 0.04 ^ef^	6.38 ± 0.02 ^abc^
	S8	7.71 ± 0.01 ^a^	6.13 ± 0.05 ^bc^
Apple (67%) and pomegranate (33%) juice samples	S9	7.49 ± 0.01 ^ef^	5.52 ± 0.24 ^e^
	S10	7.53 ± 0.01 ^de^	6.12 ± 0.04 ^bc^
	S11	7.36 ± 0.02 ^h^	5.60 ± 0.30 ^e^
	S12	7.43 ± 0.02 ^fg^	5.72 ± 0.12 ^de^

Values are means and standard errors of three determinations. Values with the different letters within one column are significantly different (*p* < 0.05).

**Table 7:** Microbial growth in fruit juices during storage (S) at room and refrigeration temperatures (in log_10_ CFU/cm^3^).

	Sample	Total Number of Aerobic Mesophilic Germs (TAMG)		Total Number of Yeasts and Molds (TYM)	
		(S at 4 °C)	(S at 20 °C)	(S at 4 °C)	(S at 20 °C)
Apple (100%) juice samples (3rd day)	S1	8.62 ± 0.01 ^cd^	8.63 ± 0.01 ^i^	8.25 ± 0.02 ^d^	7.92 ± 0.03 ^b^
	S2	8.58 ± 0.02 ^d^	8.70 ± 0.00 ^h^	8.80 ± 0.02 ^a^	8.10 ± 0.02 ^b^
	S3	8.11 ± 0.03 ^g^	8.66 ± 0.01 ^i^	7.36 ± 0.10 ^g^	7.63 ± 0.06 ^b^
	S4	8.49 ± 0.01 ^e^	8.58 ± 0.01 ^j^	7.63 ± 0.06 ^f^	7.73 ± 0.05 ^b^
Apple (67%) and pumpkin (33%) juice samples (3rd day)	S5	8.65 ± 0.01 ^c^	9.00 ± 0.00 ^c^	8.59 ± 0.01 ^b^	9.55 ± 0.01 ^a^
	S6	8.78 ± 0.00 ^a^	9.17 ± 0.00 ^a^	8.53 ± 0.01 ^bc^	9.54 ± 0.00 ^a^
	S7	8.71 ± 0.01 ^b^	8.96 ± 0.00 ^d^	8.33 ± 0.03 ^d^	9.24 ± 0.58 ^a^
	S8	8.67 ± 0.03 ^bc^	9.07 ± 0.01 ^b^	8.39 ± 0.01 ^cd^	9.58 ± 0.00 ^a^
Apple (67%) and pomegranate (33%) juice samples (3rd day)	S9	8.63 ± 0.01 ^c^	8.82 ± 0.01 ^g^	7.63 ± 0.06 ^f^	9.19 ± 0.01 ^a^
	S10	8.51 ± 0.01 ^e^	8.91 ± 0.01 ^e^	7.85 ± 0.00 ^e^	9.20 ± 0.00 ^a^
	S11	8.62 ± 0.01 ^cd^	8.86 ± 0.01 ^f^	7.52 ± 0.07 ^f^	9.30 ± 0.00 ^a^
	S12	8.42 ± 0.01 ^f^	8.85 ± 0.01 ^f^	7.36 ± 010 ^g^	9.28 ± 0.02 ^a^
Apple (100%) juice samples (6th day)	S1	9.31 ± 0.00 ^j^	9.74 ± 0.00 ^a^	7.46 ± 0.15 ^e^	10.01 ± 0.01 ^a^
	S2	9.53 ± 0.00 ^e^	9.66 ± 0.00 ^c^	6.95 ± 0.00 ^f^	9.85 ± 0.01 ^a^
	S3	9.46 ± 0.00 ^g^	9.74 ± 0.00 ^a^	6.95 ± 0.00 ^f^	8.95 ± 0.58 ^cd^
	S4	9.36 ± 0.00 ^i^	9.04 ± 0.01 ^i^	8.66 ± 0.03 ^a^	8.15 ± 0.07 ^f^
Apple (67%) and pumpkin (33%) juice samples (6th day)	S5	9.61 ± 0.01 ^c^	9.68 ± 0.00 ^b^	8.41 ± 0.02 ^b^	9.03 ± 0.01 ^cd^
	S6	9.66 ± 0.01 ^a^	9.60 ± 0.00 ^e^	7.56 ± 0.07 ^de^	9.26 ± 0.00 ^bc^
	S7	9.63 ± 0.00 ^b^	9.62 ± 0.01 ^d^	7.69 ± 0.09 ^d^	8.66 ± 0.03 ^de^
	S8	9.47 ± 0.00 ^f^	9.68 ± 0.00 ^b^	8.09 ± 0.02 ^c^	9.06 ± 0.01 ^cd^
Apple (67%) and pomegranate (33%) juice samples (6th day)	S9	9.47 ± 0.01 ^fg^	9.21 ± 0.00 ^h^	8.21 ± 0.02 ^c^	8.81 ± 0.01 ^cd^
	S10	9.58 ± 0.00 ^d^	9.66 ± 0.00 ^c^	8.18 ± 0.03 ^c^	8.84 ± 0.01 ^cd^
	S11	9.43 ± 0.00 ^h^	9.53 ± 0.00 ^f^	8.18 ± 0.03 ^c^	9.60 ± 0.00 ^ab^
	S12	9.52 ± 0.00 ^e^	9.43 ± 0.01 ^g^	8.23 ± 0.03 ^c^	8.23 ± 0.03 ^ef^
Apple (100%) Juice samples (9th day)	S1	10.06 ± 0.00 ^d^	10.68 ± 0.58 ^c^	9.73 ± 0.02 ^i^	11.02 ± 0.00 ^a^
	S2	10.39 ± 0.00 ^abcd^	10.67 ± 0.58 ^c^	10.39 ± 0.01 ^e^	11.00 ± 0.00 ^ab^
	S3	10.31 ± 0.00 ^abcd^	10.98 ± 0.00 ^a^	10.31 ± 0.01 ^f^	10.99 ± 0.00 ^b^
	S4	10.28 ± 0.58 ^bcd^	10.98 ± 0.01 ^a^	9.94 ± 0.00 ^h^	10.98 ± 0.00 ^b^
Apple (67%) and pumpkin (33%) juice samples (9th day)	S5	10.95 ± 0.00 ^a^	10.95 ± 0.00 ^a^	10.58 ± 0.00 ^d^	10.65 ± 0.00 ^h^
	S6	10.99 ± 0.00 ^a^	10.99 ± 0.01 ^a^	10.88 ± 0.00 ^a^	10.93 ± 0.00 ^c^
	S7	10.22 ± 0.00 ^cd^	10.35 ± 0.56 ^d^	10.22 ± 0.01 ^g^	11.03 ± 0.00 ^a^
	S8	10.22 ± 0.00 ^cd^	11.03 ± 0.57 ^a^	10.22 ± 0.00 ^g^	10.71 ± 0.00 ^g^
Apple (67%) and pomegranate (33%) juice samples (9th day)	S9	10.61 ± 0.58 ^abcd^	10.75 ± 0.00 ^b^	10.65 ± 0.01 ^c^	10.74 ± 0.03 ^f^
	S10	10.52 ± 0.01 ^abcd^	10.98 ± 0.00 ^a^	10.52 ± 0.01 ^d^	10.99 ± 0.00 ^b^
	S11	10.84 ± 0.00 ^abc^	10.84 ± 0.01 ^b^	10.84 ± 0.00 ^b^	10.85 ± 0.00 ^d^
	S12	10.83 ± 0.00 ^abc^	10.89 ± 0.01 ^b^	10.72 ± 0.00 ^c^	10.79 ± 0.00 ^e^

Values are means and standard errors of three determinations (considering one S1–S12 data group on each day separately). Values with the different letters within one column per each day of determination are significantly different (*p* < 0.05).

## Data Availability

The data used to support the findings of this study can be made available by the corresponding author upon request.
